# Speciation and Luminescence of a Binuclear Lanthanide Complex Bearing an Aminophenolate Chromophore

**DOI:** 10.1002/chem.202502305

**Published:** 2025-08-29

**Authors:** Villads R. M. Nielsen, Charlie H. Simms, Daniel Kovacs, Matthew F. Allen, Matthew J. Langton, Stephen Faulkner, Thomas Just Sørensen

**Affiliations:** ^1^ Department of Chemistry and Nano‐Science Centre University of Copenhagen Universitetsparken 5 København 2100 Denmark; ^2^ Department of Chemistry Chemistry Research Laboratory Oxford University Mansfield Road Oxford OX1 3TA UK

**Keywords:** coordination chemistry, lanthanide luminescence, luminescent probes, solution chemistry, speciation

## Abstract

Lanthanide(III) complexes show diverse speciation in solution. Here, the photophysical properties and speciation in solution of binuclear lanthanide complexes bearing an aminophenolate chromophore that reversibly bind to both metal centers were determined. The aminophenolate can reversibly bind to both lanthanide ions in a pH dependent manner. Above pH 3 the phenolate moiety remains deprotonated and the lanthanide complexes exhibit very high kinetic stability, however, protonation of the amino group modulates the luminescence intensity without changing the luminescence lifetime of the lanthanide. Below pH 3, protonation of the phenol results in a flexible, open structure, leading to reversible demetallation of the lanthanide complex as the system comes under thermodynamic control. This study highlights the importance of a detailed understanding of both the speciation and pH responsive behavior of highly luminescent binuclear lanthanide complexes for their development as responsive probes. These results confirm the promise of phenolate derived binuclear complexes as highly luminescent responsive probes, modulated by changes in pH.

## Graphical abstract

Lanthanide complexes are used in patients and for bioimaging and assays. The solution structure and speciation are critical when evaluating function, and here we fully unravel the structure and photophysics of a binuclear lanthanide complex.

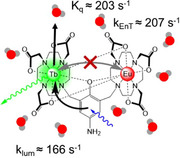

## 1 Introduction

Multinuclear lanthanide complexes have underpinned some of the most significant advances in f‐element chemistry in recent years.^[^ ^1^ ^]^ In particular they have shown great promise in the development of high temperature single molecule magnets (SMM's),^[^ ^2^ ^]^ as responsive units in responsive molecules,^[^ ^3^ ^]^ and displaying an enhanced luminescent output.^[^ ^4^ ^]^ The presence of two or more lanthanide centers enables ratiometric readouts,^[^ ^5^ ^]^ white light generation,^[^ ^6^ ^]^ and the exploitation of the unique properties of several lanthanide ions, with the potential for dual probes to be recognized, combing the impressive magnetic and luminescent properties of lanthanides.^[^ ^7^ ^]^

Lanthanide coordination chemistry involves delicate interplay between competing equilibria, and lanthanide luminescence is the result of a subtle competition between rates.^[^ ^8^ ^]^ The stability of lanthanide complexes is defined by the free energy of the relevant species in solution, trivial in simple solutions,^[^ ^9^ ^]^ yet complex when competing ions such as protons, copper, magnesium, and zinc are present. Cyclen‐derived ligands with eight donor atoms generally form kinetically inert complexes, while analogues with seven donor atoms may form kinetically labile complexes.^[^ ^8^ ^]^

Making multinuclear lanthanide complexes poses some synthetic challenges, but these can be overcome using kinetically inert building blocks, enabling the linear synthesis or multicomponent reactions that result in highly complicated multinuclear lanthanide complexes.^[^ ^8^ ^]^ Moreover, there are only a few reported syntheses of heteronuclear complexes, each with elegant but complex synthetic procedures.^[^ ^8^, ^10^ ^]^

Previous studies which investigate the optical spectroscopy of multinuclear Ln(III) complexes revealed that additional photophysical processes become feasible, including the energy transfer between individual Ln(III) centers, providing there is sufficient spectral overlap between the two ions.^[^ ^11^ ^]^ This energy transfer is dependent on the energy levels of the cations, and the distance between them in solution. Moreover, this interaction can be mediated by changes in the local environment, such as changes in pH, which can modulate existing energy transfer pathways and even introduce new electron transfer pathways.^[^ ^12^ ^]^ Importantly, changes in pH can also induce changes in the inner sphere coordination of the Ln(III) center, where bridging or chelating ligands can reversibly coordinate to the Ln(III) center upon deprotonation.^[^ ^13^ ^]^ Therefore, by understanding the speciation and structures in solution, vital information about the behavior and physical properties of multinuclear Ln(III) systems, can be understood in detail.

**Scheme 1:**
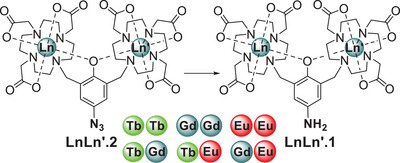
Investigated homometallic and heterometallic lanthanide(III) complexes.

Here, we unravel the speciation and excited state properties of the multinuclear lanthanide complex [Ln_2_ **.1**]^−^ (Scheme 1) made from a ligand with two DO3A (1,4,7,11‐tetraazadodecane‐1,4,7‐triacetic acetate) lanthanide binding pockets grafted at the benzylic carbon atoms on a 2,6‐dimethyl‐*p*‐aminophenol using both optical spectroscopy and NMR, where by both the aniline and the phenol are expected to exist as ammonium and phenolate ions in a pH dependent manner. Although there are numerous investigations into the optical properties of kinetically stable binuclear Ln(III) complexes, there are only a handful of examples that present the corresponding investigations in ^1^H NMR.^[^ ^14^ ^]^

This system, and a variety of similar systems, have been studied previously.^[^ ^5^, ^14^, ^15^ ^]^ The reduction of a related complex to [Tb_2_ **.1**]^−^ exhibits an impressive luminescent probe, and the analogous reduction leading to [Gd_2_ **.1**]^−^ acts as a MR probe, both for the sensing of redox environments.^[^ ^16^ ^]^ Particularly, these compounds show promise for investigating hypoxic environments such as tumour cells, however it is important to note that these hypoxic tumour microenvironments often tend to be acidic.^[^ ^17^ ^]^ In order for these systems to be applied in vivo the pH stability, speciation and properties of these species must be investigated in detail.

In this study, the speciation and luminescence are determined in unprecedented detail, showing that the complex dissociates at low pH, exhibiting very limited intermetallic excited state energy transfer, and forms highly luminescent terbium(III) complexes. Additionally, the synthesis reported, for the formation of heterobinuclear complexes, is a simple but effective stoichiometric approach which could be applied to any binuclear system with two or more identical coordination sites.

## 2 Results and Discussion

Ln_2_.**1** was prepared in situ by reduction of Ln_2_ **.2**, an azidophenol derivative, as shown in Scheme 1, see the Supporting Information for details. In addition to the homonuclear complexes Ln_2_ **.1** (Ln = Gd^III^, Tb^III^, and Eu^III^), heteronuclear versions, LnLn’**.1**, were also prepared with Gd^III^Tb^III^, Eu^III^Tb^III^, Gd^III^Eu^III^. These heterometallic systems exploit the kinetic stability of the mononuclear system [LnH_3_ **.1**]^−^, which was prepared by HPLC purification of a statistical mixture resulting from the addition of one equivalent of lanthanide‐triflate (Ln(OTf)_3_) to the proligand, all details and compound characterisation are provided as Supporting Information.

**Figure 1:**
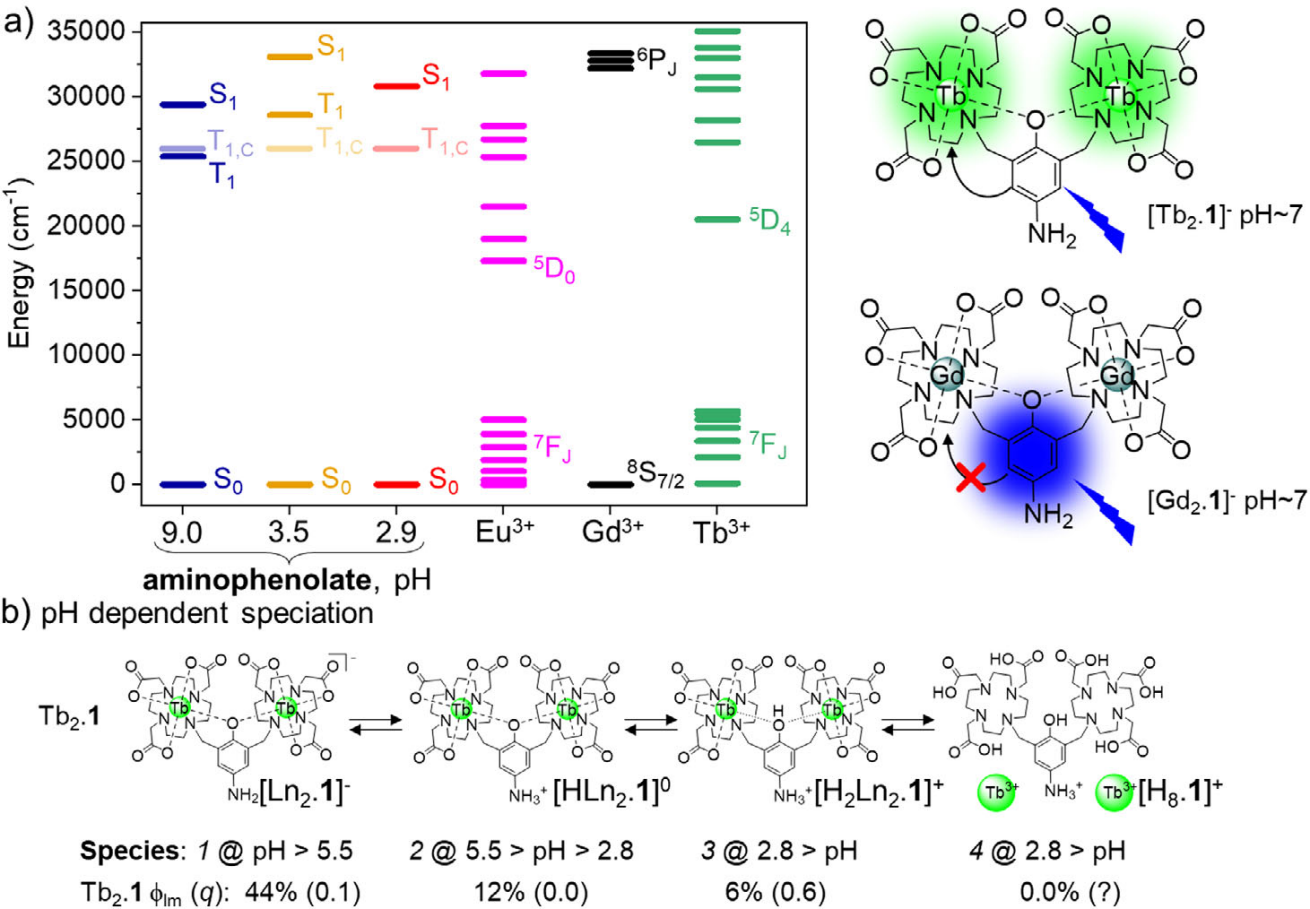
a) Energy level diagram for Ln_2_.**1** and LnLn’.**1** with Ln = Eu, Tb and Gd as a function of pH. b) Structure of Tb_2_.**1** and Gd_2_.**1** and an illustration of the outcomes upon excitation of the aromatic chromophore within the ligand. c) pH dependent speciation of Ln_2_.**1** illustrated using Tb_2_.**1** with the quantum yield of terbium‐centered luminescence (ϕ_Tb_) and number of inner sphere solvent molecules (*q*) indicated.

The Gd^III^ complexes were prepared as model systems to explore the heavy atom and paramagnetic effects on the aminophenol photophysics,^[^ ^18^ ^]^ without the option of excited state energy transfer from the aminophenol to a lanthanide center as shown by the energy diagram in Figure 1. As has been previously reported, [Tb_2_.**1**]^−^ exhibits luminescence from the terbium center with a high quantum yield (44%).^[^ ^16^ ^]^ However, while the nitrophenol derivative has been shown to be luminescent in previous work,^[^ ^14a^ ^]^ initial studies showed that no europium luminescence could be observed following excitation into the ligand excited state of the aminophenol derivatives [Eu_2_.**1**]^−^ or [TbEu.**1**]^−^. Closer scrutiny revealed a very weak europium luminescence, see Figure S30.

**Figure 2:**
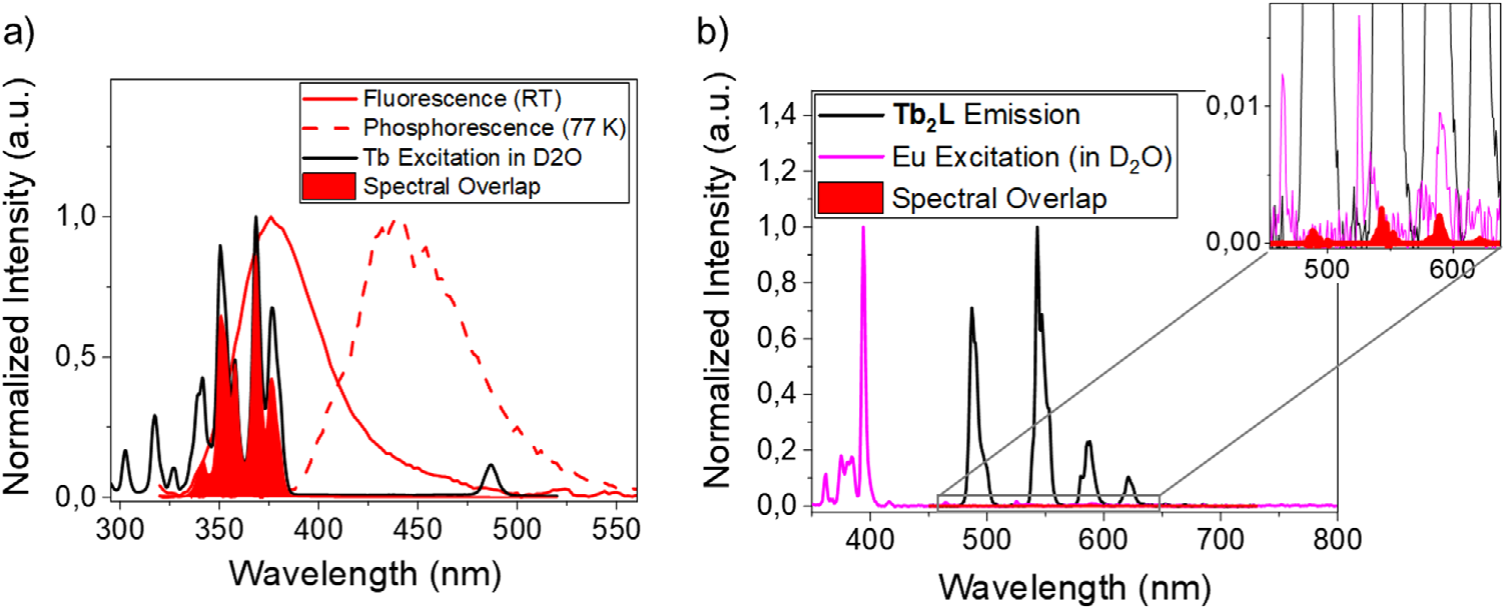
a) [Tb(D_2_O)_9_]^3+^ excitation (em = 545 nm) spectrum superimposed on the aminophenolate fluorescence and phosphorescence spectrum from [Gd_2_.**1**]^−^. The calculated spectral overlap between the aminophonelate singlet excited state S_1_ and the [Tb(D_2_O)_9_]^3+^ excited state manifold is shown in solid red. Note that the carboxylate phosphorescence is not shown b) [Eu(D_2_O)_9_]^3+^ excitation (em = 700 nm) spectrum superimposed on the terbium luminescence spectrum from [Tb_2_.**1**]^−^. The calculated spectral overlap between the terbium centred ^5^D_4_ luminescence and the [Eu(D_2_O)_9_]^3+^ excited state manifold is shown in solid red; only visible in the magnified insert.

As terbium luminescence was the only strong luminescence observed, we focused on investigating the luminescent properties of [Tb_2_.**1**]^−^ and [TbEu.**1**]^−^. There is negligible overlap between transitions present in terbium emission and europium absorption spectrum (Figure 2a), suggesting that energy transfer from the ^5^D_4_ multiplet of Tb(III) to the ^5^D_0_ multiplet of Eu(III) is unlikely to be efficient. However, as shown in Figure 1a the ^5^D_3_ multiplet of Tb(III) overlaps with a number of excited states in the upper manifold of the europium energy level diagram, suggesting that energy transfer to these states is feasible. The energies of these Eu(III) centered states are very similar to that of the ligand triplet state (T_1_), which can also equilibrate with these intermediate excited states. As such, energy transfer to the europium manifold is likely to result in quenching via the ligand triplet and europium‐to‐ligand redox states, resulting from photoinduced electron transfer (PET). To contrast, Figure 2 describes both the overlap required for ligand‐to‐terbium and terbium‐to‐europium energy transfer, and it is clearly seen that the difference in overlap—and thus the difference in the rates of these two energy processes—is significant.^[^ ^19^ ^]^ By comparison of the lifetimes of [TbGd.**1**]^−^ and [TbEu.**1**]^−^, we can determine the rate of terbium(^5^D_4_)‐to‐europium energy transfer is 200 s^−1^, see Supporting Information for details. This is comparable to the rate of energy transfer to a single water molecule, but is slow compared to an expected rate of PET.^[^ ^20^ ^]^ However, there is a dramatic difference in emissive quantum yield between [TbEu.**1**]^−^ (0.3%) and [Tb_2_.**1**]^−^ (44%), while the quantum yield for [TbGd.**1**]^−^ (47%) is comparable to that of [Tb_2_.**1**]^−^. These quantum yields confirm that the excited state manifold can quench the observed emission from terbium despite the small change in emissive lifetime of ^5^D_4_ from τ_Tb_([TbGd.**1**]^−^) = 2.5 ms to τ_Tb_([TbEu.**1**]^−^) = 1.7 ms. Thus, the hypothesis is that energy equilibrate in the upper manifold before the terbium ^5^D_4_ state can form.

**Figure 3:**
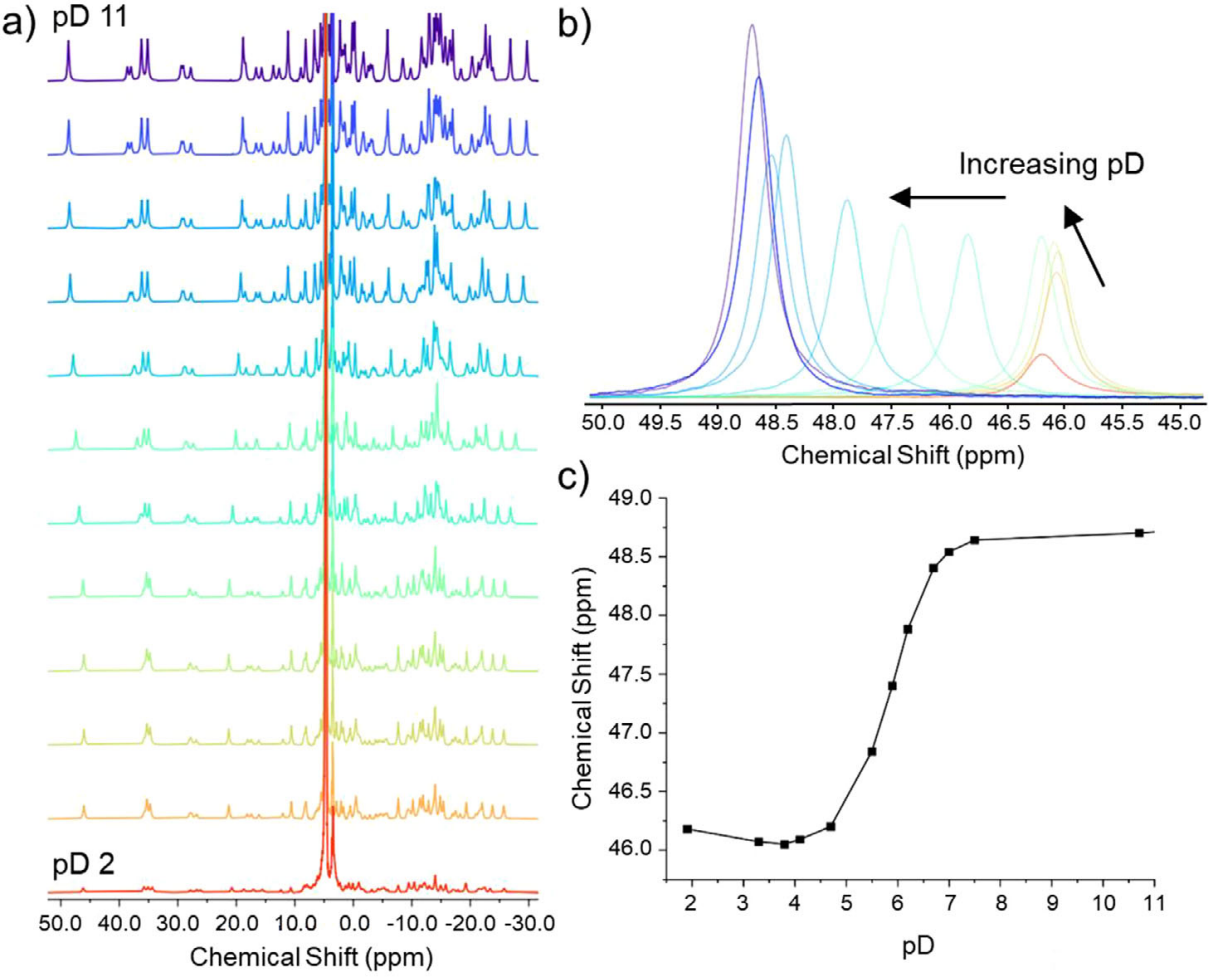
a) Paramagnetic NMR pD titration using Eu_2_.1 from pD 2.0 (bottom) to pD 11 (top). b) Overlay showing the change in the axial protons on the cyclen rings from pD 2.0 to pD 11.0 c) Titration isotherm following the chemical shift of the axial protons as a function of pD.

**Figure 4:**
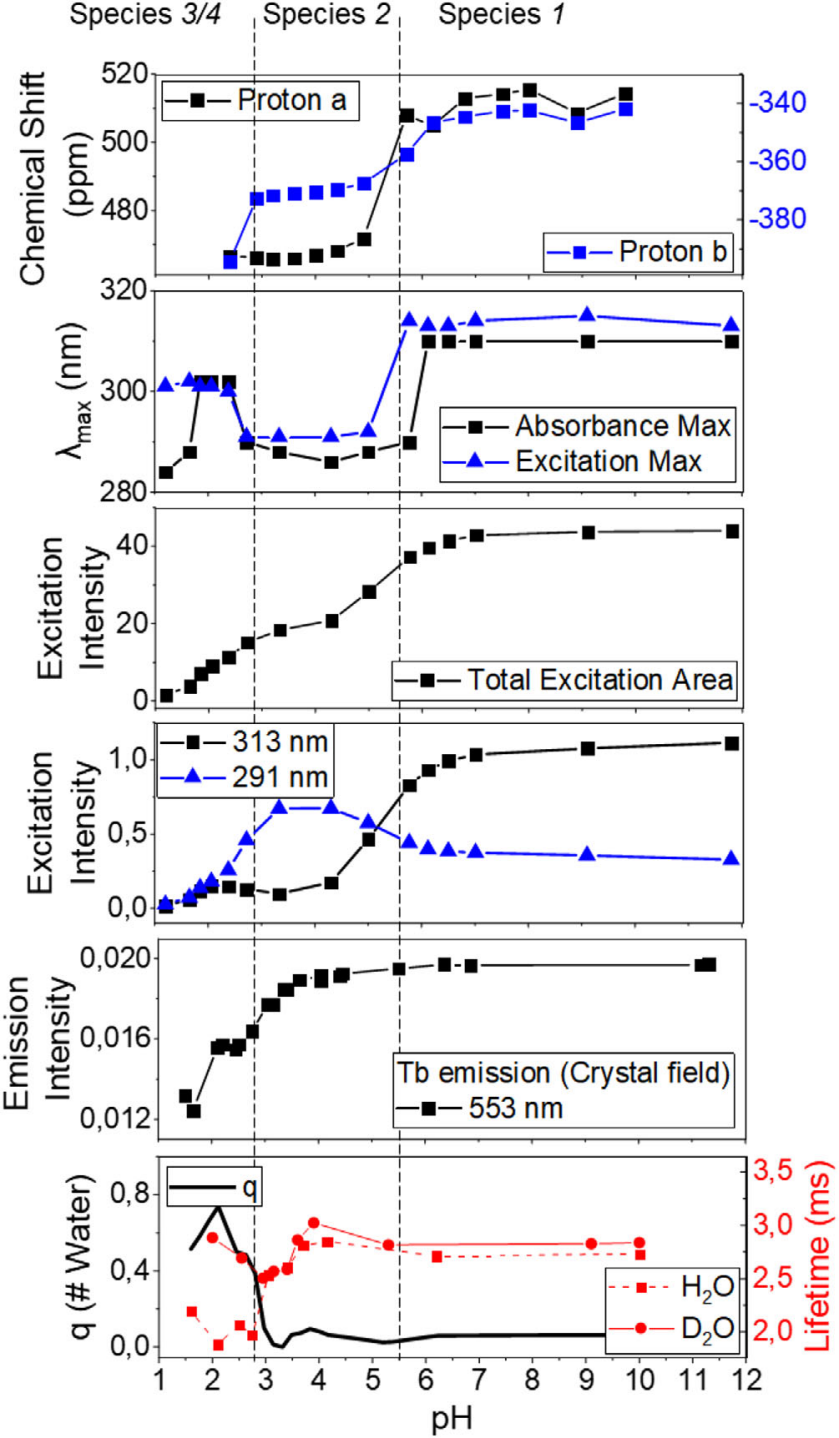
Titration isotherms following the change in speciation of [Tb_2_ **.1**]^−^ as a function of pH/pD. The regions where species *1*(pH > 6.8), *2*(6.8 > pH > 2.8), and *3*/*4*(2.8 > pH) dominate is indicated with dashed lines across all isotherms. From top to bottom: Chemical shift of axial proton on cyclen ring (a) and acetate methylene protons (b), raw data in Supporting Information. Absorption and excitation spectrum maximum λ_max_ (main band). Total area of the excitation spectrum. Intensity recorded in the excitation spectrum at the aminophenolate band (313 nm) and the ammoniumphenolate band (291 nm). Emission intensity in a specific line (553 nm) of the ^5^D_4_ → ^7^F_6_ emission band (545 nm). Luminescence lifetime of the ^5^D_4_ multiplet in Tb(III) in H_2_O and D_2_O with the number of inner sphere water molecules *q*.

The aminophenol backbone of [Ln_2_.**1**]^−^ has two pH active groups. The phenol with a p*K*_a_ of 10.2 and the aniline with a p*K*_a_ of 5.3 in the free ligand that is without the appended Ln.DO3A groups.^[^ ^21^ ^]^ Binding of the lanthanide(III) ions will alter the apparent p*K*_a_ values, as the phenol lone pairs are involved in coordinating the lanthanide(III) ions, which in turn reduce the electron density on the aniline nitrogen. Thus, we expected to see two pH dependent events in the speciation of [Ln_2_.**1**]^−^ involving the three species shown in Figure 1b: [Ln_2_.**1**]^−^, [HLn_2_.**1**]^0^ and [H_2_Ln_2_.**1**]^+^. Only two of these species were observed by NMR. Figure 3 shows the paramagnetic NMR of [Eu_2_ **.1**]^−^ in water, changing pD we observe a single change to the ligand structure from [Eu_2_ **.1**]^−^ to [HLn_2_.**1**]^0^. Following a single resonance, in Figure 3 the most shifted resonance of the axial protons on the DO3A binding pockets, we can determine that the change occur with pKa around 6. Assigning this event to protonation of a specific site is not possible from the NMR alone, as the resonances that can be identified are far from the protonation sites. The luminescence data is richer, as lanthanide luminescence informs on the number of solvent molecules bound to the lanthanide center *q*, the coordination geometry, and the energy transfer cascade that gives rise to emission from the lanthanide. In addition to the luminescence data, the ligand absorption is also pH dependent. Nevertheless, we investigated both the [Eu_2_ **.1**]^−^ and [Tb_2_ **.1**]^−^ complexes using NMR. The data from [Tb_2_ **.1**]^−^ are supplied as Supporting Information. The isotherms following the axial (around ‐360 ppm) and acetate protons (around 490 ppm) are shown in Figure 4.

Figure 4 shows how the experimental observables of [Tb_2_ **.1**]^−^ change as a function of pH. From the top, the ^1^H NMR indicates two species, but when comparing the six panels in Figure 4 that include absorption and excitation data it becomes evident that four species, as shown in Figure 1b, must be considered. At neutral to high pH (> 5.5) [Tb_2_.**1**]^−^ is present as the deprotonated form of the complex with no water molecules in the lanthanide coordination sphere (q = 0), where q represents the number of bound solvent, as determined by the Horrocks equation (see equation S1).^[^ ^22^ ^]^ At weakly acidic pH (6 > pH > 2.8), the aniline is protonated forming [HTb_2_.**1**]^0^. This can be seen in the changes observed in both the ligand ^1^H NMR and absorption spectra, however, the data implies no change in the local coordination around the lanthanide center (q = 0). At acidic pH (<2.8) the phenol is protonated forming [H_2_Tb_2_.**1**]^+^, a metastable species that undergoes decomplexation over a period of hours to form [H_8_.**1**]^+^ and two free Tb^III^ ions, see below for details.

The change from [H_2_Ln_2_.**1**]^+^ to [H_8_.**1**]^+^ is readily assigned as the free ions cannot be excited through the ligand. And the change from [HLn_2_.**1**]^0^ to [H_2_Ln_2_.**1**]^+^ is readily identified as the protonation of the phenol, which changes the coordination sphere of Tb(III). This is evidenced by slight, but significant, changes to the crystal field, as seen in the emission band (em = 553 nm) and by an increase in *q* from 0 to 0.5, indicating the presence of a single OH group in close proximity to the Tb(III) ions. Neither of these changes are observed at pH > 4. Thus, the identity of the two first species must differ in the ligand alone, i.e., the aniline [Tb_2_ **.1**]^−^ and anilinium [HTb_2_.**1**]° form, respectively. This leads to the conclusion that in [Tb_2_ **.1**]^−^ the p*K*_a_ of the phenol and the aniline moieties are reversed. The phenol p*K*_a_ shifts from 10 to 2.8 and the anline p*K*_a_ shifts from 5.3 to 5.5 when the phenolate oxygen is coordinated to two DO3A groups with electropositive lanthanide ions. Perhaps more surprisingly, the complex is unstable below pH ∼3. Many lanthanide containing systems (including Ln.DOTA) are known to exhibit pH dependent dissociation kinetics.^[^ ^7a^ ^]^ In the case of the aminophenolate system, the notable feature is the step change in stability following the protonation of the phenol oxygen.

Having identified and assigned the [Tb_2_.**1**]^−^, [HTb_2_.**1**]^0^, and [H_2_Tb_2_.**1**]^+^ species we can assign the experimental properties shown in Figure 4. [Tb_2_.**1**]^−^ is species 1 with the highest paramagnetic shift of all protons, most redshifted chromophore absorption, and highest emission intensity. The latter is due to the lack of quenching by inner sphere O‐H oscillators, which is shown by a *q*‐value of zero. In [HTb_2_.**1**]^0^ *q* remains zero when the aniline is protonated, yet the more electropositive anilinium group changes the conformation of the complex, which reduces the paramagnetic shift observed in ^1^H NMR, blueshift the chromophore absorption from 313 nm to 291 nm, but does not change the crystal field experienced by the Tb^III^ centres. In the final complex [H_2_Tb_2_.**1**]^+^ the properties change dramatically as the protonation of the phenol changes the entire structure of the complex, *q* rises toward 0.5 and the crystal field changes. However [H_2_Tb_2_.**1**]^+^only exist transiently, and the sample will be a mixture of [H_2_Tb_2_.**1**]^+^, [H_8_.**1**]^+^ and [Tb(H_2_O)_9_]^3+^.

**Figure 5:**
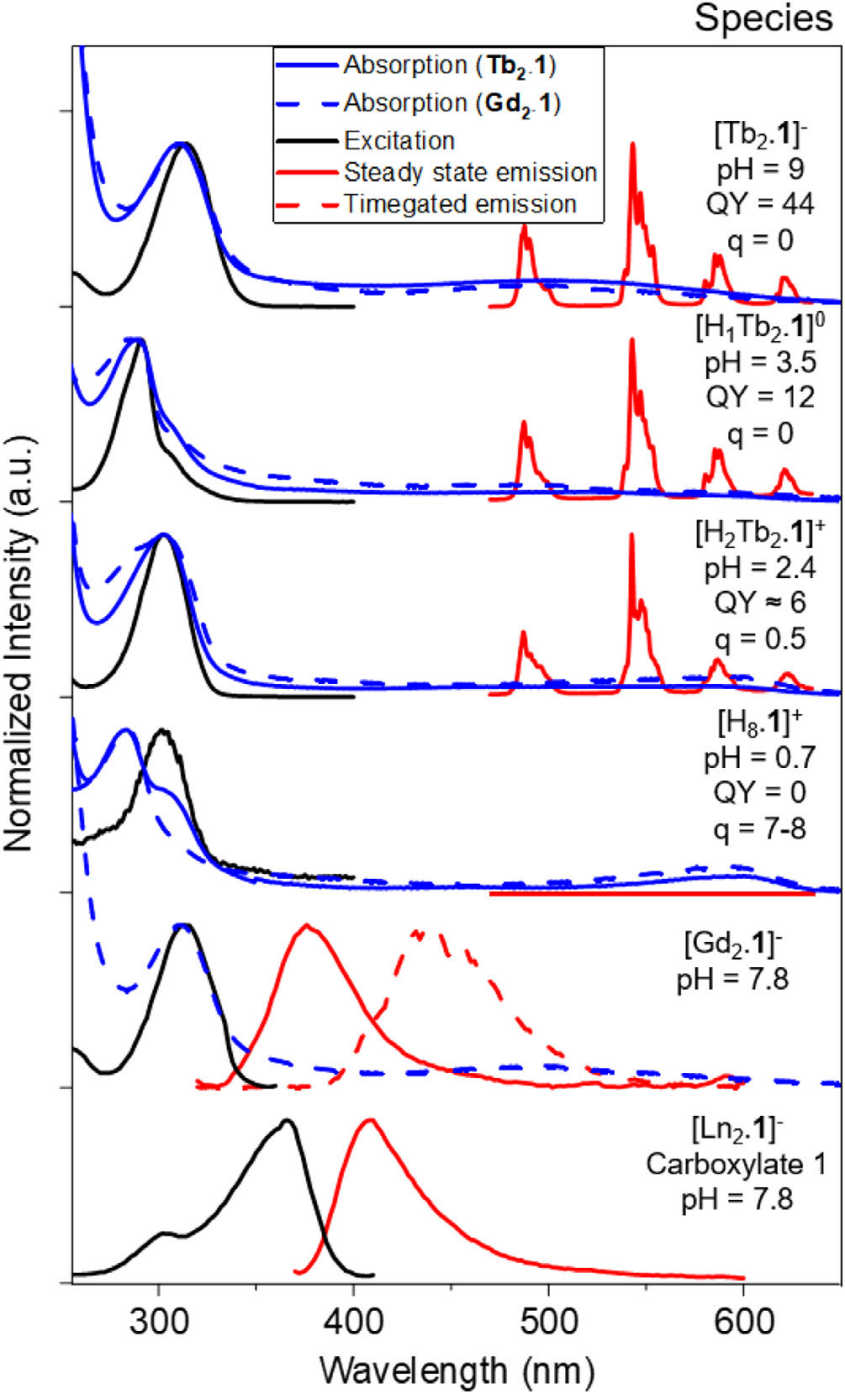
Luminescence and absorption spectra of **Ln_2_.1** as a function of pH. The spectra show the absorption, emission and excitation spectra of the Tb(III) center, the aminophenolate chromophore and carboxylate chromophores. QY = Quantum yield, *q* = number of inner sphere water molecules.

The four species can also be identified from the pH dependence of the photophysical properties of the complexes. The data is shown in Figure 5 and tabularized in Table S1. Upon protonation at the aniline, the quantum yield of emission falls from 44% for [Tb_2_ **.1**]^−^ to 12% for [HTb_2_.**1**]^0^. Below pH 3, the quantum yield falls to 6%, but slowly decrease toward zero with time as the complex dissociates.

The absorption, excitation, and emission spectra of the Ln_2_.**1** species are shown in Figure 5 where the photophysical data are also provided. To understand the photophysics it is important to note that Ln_2_.**1** consists of multiple distinct units with localised excited states: the aminophenolate chromophore, the carboxylate groups of the DO3A binding pockets, and the Ln(III) centres.

The predominate change for the Tb(III) photophysics in Tb_2_.**1** is the observed quenching of the terbium luminescence resulting from the presence O‐H oscillators. The second important change in the photophysical properties arise from changes in the excited state energy transfer cascade that leads to sensitised terbium luminescence. The inner sphere hydration at the metal centers *q* can be determined using the modified Horrocks equation,^[^ ^20^, ^22^, ^23^ ^]^ confirming that the closed conformer of the complexes exists above pH 3 (since q = 0) in both [Tb_2_.**1**]^−^ and [HTb_2_.**1**]^0^, while the initial [H_2_Tb_2_.**1**]^+^ species formed below pH 3 exhibits q = 0.5, consistent with protonation of the phenol. Considering the aminophenolate chromophore, three distinct electronic structures are formed as a function of pH: an aminophenolate, an ammoniumphenolate, and an ammoniumphenol, and the photophysical properties change across these species. Cursory inspection of Figure 5 shows that this is reflected in both the excitation spectra monitoring the lanthanide luminescence and the absorption spectra. Finally, time‐resolved emission of [Gd_2_.**1**]^−^ and [HGd_2_.**1**]^0^ allowed for mapping of the triplet states in the aminophenolate chromophore. The energies of the relevant aminophenol chromophore states are shown in Figure 1 and tabulated in the Supporting Information (Table S1). The last isolated electronic energy levels are found at carboxylate groups of the DO3A binding pockets. These are either bound to an electropositive lanthanide ion or a proton and in these two forms the absorption, excitation, and emission spectra does not change. Thus, the spectra of carboxylate groups in the ligand, measured at pH 7.8 and shown in Figure 5, are constant with pH and species, see Supporting Information for details. Note that these carboxylate centred electronic states are always present in cyclen‐derived—and all other polyaminocarboxylate—ligands.^[^ ^24^ ^]^

**Figure 6:**
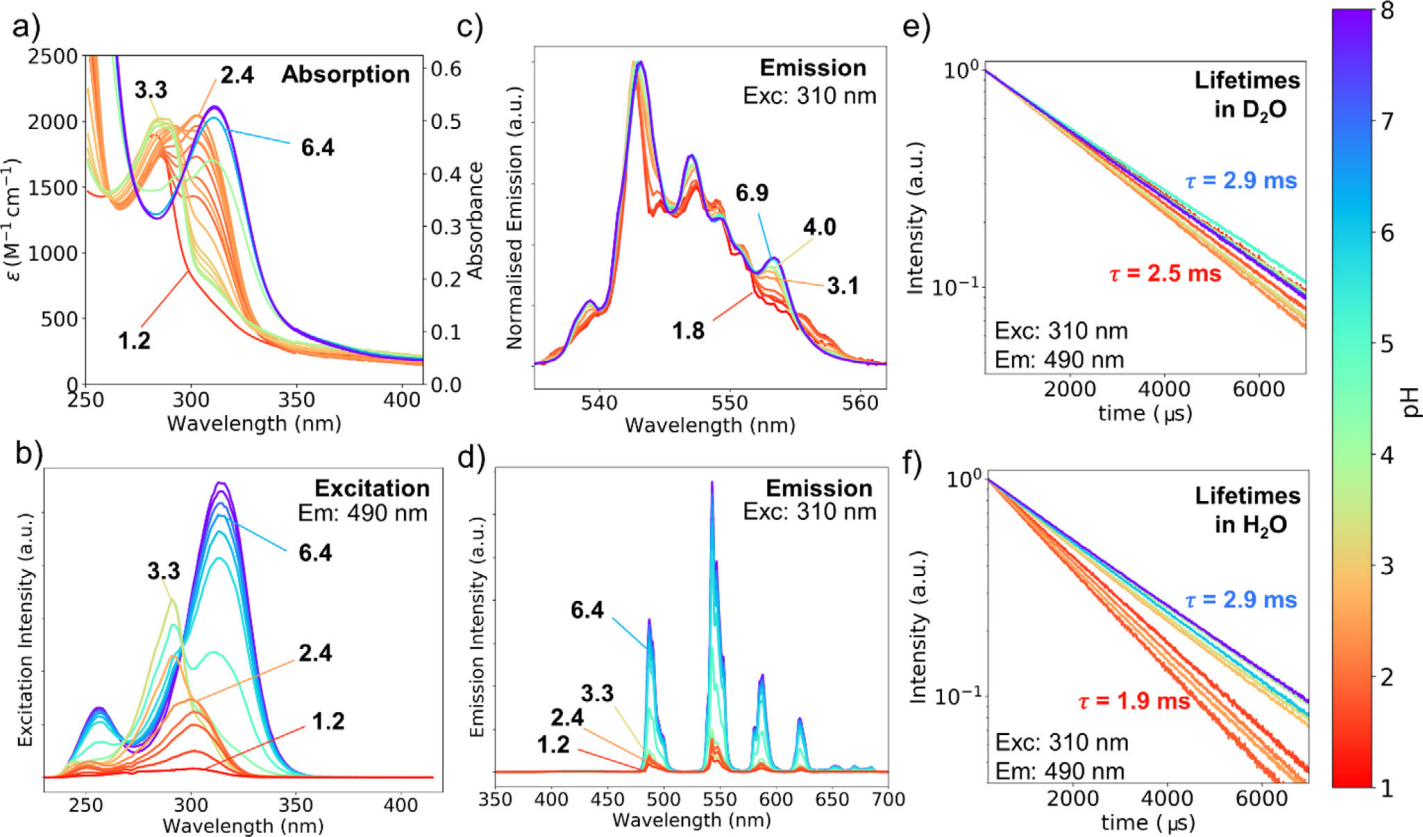
Effects of pH on the photophysical data obtained from **Tb_2_.1**. a) Changes in the main absorption band. b) Changes in the steady‐state excitation spectrum monitoring emission in the ^5^D_4_ → ^7^F_6_ band at 490 nm. c) Changes in the steady‐state emission spectrum following excitation in the aminophenolate chromophore at 310 nm. d) Changes in the crystal field splitting of the ^5^D_4_ → ^7^F_5_ band at 545 nm monitored using the steady‐state emission spectrum following excitation in the aminophenolate chromophore at 310 nm. e) Time‐resolved emission decay profiles monitored at 490 nm following excitation at 310 nm in the aminophenolate chromophore in H_2_O and D_2_O.

Where Figure 5 shows the spectra and photophysical properties of [Tb_2_.**1**]^−^, [HTb_2_.**1**]^0^ and [H_2_Tb_2_.**1**]^+^, Figure 6 shows how spectra and lifetime changes as a function of pH. Figure 6a shows the ligand centered absorption band change as a function of pH, detailing the change shown in figure 5 and discussed above. The change in the absorption spectra (Figure 6a) is reflected in the excitation spectra recorded monitoring the lanthanide luminescence, which furthermore reports on the energy transfer efficiency (Figure 6b). As the [H_8_.**1**]^+^ species is formed, the ligand‐to‐lanthanide energy transfer is switched off, and the ligand absorption disappears from the excitation spectra. The terbium luminescence changes as a function of pH, figure 6c shows that the changes can be followed upon monitoring the crystal field changes, which report on the changes in the coordination geometry around each Tb(III) center. In the crystal field the change is most prominent upon protonation of the phenolate, and when demetallation occurs and [Tb(H_2_O)_9_]^3+^ forms. Figure 6d shows that the changes can also be monitored in the total emission intensity, which mirrors the excitation spectra in Figure 6b. Note that the intensity change can be tailored to increase with pH by exciting at, e.g., 330 nm or decrease with pH by exciting at, e.g., 275 nm. The terbium luminescence can also be probed using the luminescence lifetimes. Data in D_2_O (Figure 6e) and H_2_O (Figure 6f) indicate the presence of quenching water molecules in the inner coordination sphere of lanthanide complexes,^[^ ^20^, ^22^, ^23^ ^]^ major changes are only observed below pH 3. The data in Figure 6 details the speciation determined in Figure 5, and by combining the data in Figure 5 and Figure 6 we are able to determine the excited state manifold of Ln_2_.**1** shown in Figure 1.

**Figure 7:**
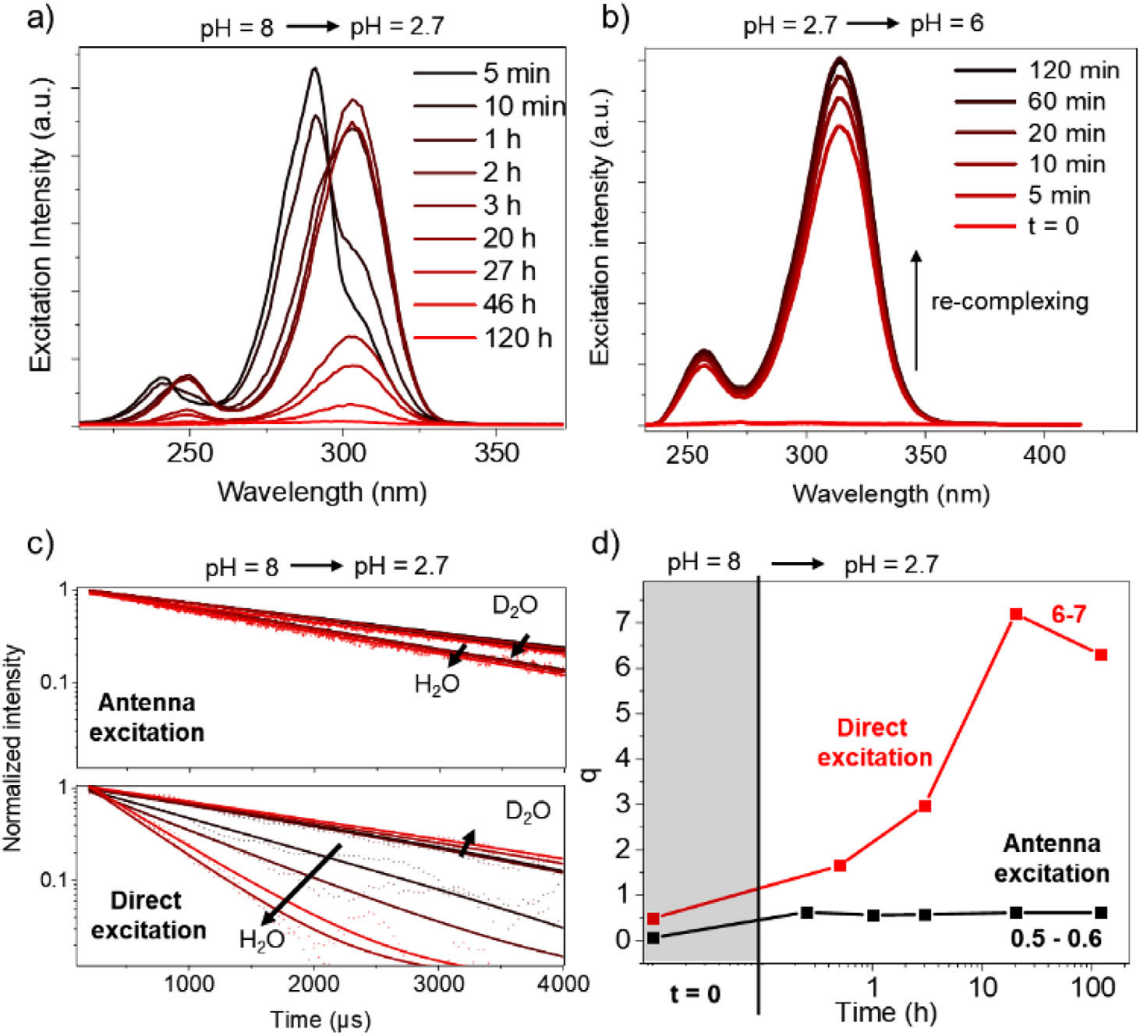
Monitoring the decomplexation of **Tb_2_.1** at pH < 2.8 using photophysical parameters. a) Changes in the steady‐state excitation spectrum monitoring emission in the ^5^D_4_ → ^7^F_6_ band at 490 nm at pH 2.7 as a function of time from 5 minutes to 120 hours. b) Changes in the steady‐state excitation spectrum monitoring emission in the ^5^D_4_ → ^7^F_6_ band at 490 nm at pH 6 as a function of time from 5 to 120 minutes. c) Changes in observed luminescence lifetime of ^5^D_4_ following excitation in the aminophenolate chromophore at 310 nm and following excitation in the ^7^F_6_ → ^5^D_4_ band at 490 nm. d) Changes in the number of inner sphere water molecules *q* when calculated using direct and through ligand excitation.

To quantify the rate of decomplexation that followed the protonation of the phenolate, i.e., at pH < 3, we performed a kinetic experiment following the Tb(III) ion decomplexation induced by reduction in pH. Figure 7a shows that the decomplexation occurs over 120 hours at low pH. Upon subsequently increasing the pH complete recomplexation is observed within 120 minutes (Figure 7b). By comparison of both the luminescence lifetimes (Figure 7c) and the resulting *q*‐values (Figure 7d) following antenna excitation via the aminophenolate chromophore (λ_ex_ = 310 nm) or following direct excitation of the ^7^F_6_ → ^5^D_4_ transition in Tb^III^ (λ_ex_ = 490 nm) it is clear that a highly hydrated system is formed at below pH < 3 as *q* was found to be larger than 6. As the emission intensity of [Tb(H_2_O)_9_]^3+^ is orders of magnitude lower than that of [H_2_Tb_2_.**1**]^+^ following excitation at 310 nm, the free Tb(III) ions—and the true speciation of Tb(III) in the samples—are only detectable upon direct excitation of Tb(III). As the complex is the dominating species in the data when the sample is observed following aminophenolate excitation, conclusions drawn without comparing to direct excitation risk being wrong. Both direct and sensitized spectra should always be recorded, if speciation is relevant.^[^ ^25^ ^]^

## 3 Conclusion

A multinuclear lanthanide complex [Ln_2_.**1**]^−^ (Ln = Tb, Gd, Eu) and its heterometallic analogues [TbLn.**1**]^+^ (Ln = Gd, Eu) were prepared and their speciation, photophysical properties and the systems pH dependence were investigated in detail. We conclude that direct terbium‐to‐europium excited state energy transfer is not favourable in this system. These complexes are kinetically stable over a broad pH range (including around physiological pH), but become labile under moderately acidic conditions (pH < 3). Since they readily reform if pH is increased above 3, we can infer that the system is under thermodynamic control below pH 3 and under kinetic control above pH 3. Such considerations of potential decomplexation pathways are important for any system that might be applied in vivo, and it is clear from these results that the behaviour of all pendent groups on cyclen derivatives need to be taken into account when designing ligand structures. Even at very low pH, the slow rates of metal release observed here are consistent with synthetic manipulations on the timescale of many chemical reactions.

Despite the range of protonation states available to the aminophenolate backbone used here, the [Tb_2_.**1**]^−^ complex was found to have a high quantum yield, with predictable speciation around physiological pH. Phenol derived bicyclic systems such as these have clear potential as luminescent lanthanide‐based probes, excluding water from the inner coordination sphere and providing a tuneable chromophore that can be matched to a variety of lanthanide ions and future applications.

## Supporting Information

The authors have cited additional references within the Supporting Information.^[^ ^7^, ^16^, ^22^, ^26^ ^]^

## Conflict of Interest

The author declare no conflict of interest.

## Supporting information



Supporting Information

## Data Availability

The data that support the findings of this study are available in the supplementary material of this article.
