# Supplementary material for: Speciation and Luminescence of a Binuclear Lanthanide Complex Bearing an Aminophenolate Chromophore
Source: Chemistry. 2025 Aug 29;31(56):e02305. doi: 10.1002/chem.202502305 (PMC12510142; doi:10.1002/chem.202502305)
Supplement: Supplementary file 1 — Supporting Information [file CHEM-31-e02305-s001.pdf]

Supplementary Information for:

Speciation and luminescence of a binuclear lanthanide complex bearing an aminophenolate chromophore

Villads R. M. Nielsen[†a,b], Charlie H. Simms[†b], Daniel Kovacs[b], Matthew F. Allen[b], Matthew J. Langton[b], Stephen Faulkner[b]*, and Thomas Just Sørensen[a]*

[†] These authors contributed equally.

[a] *Department of Chemistry and NanoScience Centre, University of Copenhagen, Universitetsparken 5, 2100 Copenhagen, Denmark, TJS@chem.ku.dk.*

[b] *Department of Chemistry, University of Oxford, Chemistry Research Laboratory, Mansfield Road, Oxford, OX1 3TA, United Kingdom, stephen.faulkner@keble.ox.ac.uk.*

Contents

Contents S2

Synthesis & Characterisation S3

- Synthesis and Purification S3
- Characterisation Information S3
  - Mass Spectrometry S3
  - NMR spectrometry S3
- Synthesis of Bimetallic Complexes S4
- Synthesis of Heterobimetallic Complexes S6
  - Preparation of the monometallic complexes S6
  - Preparation of the heterobinuclear complexes S7
  - Example of reduction of Heterobimetallic complexes S7

Investigation of Speciation S8

- 1H NMR spectroscopy S8
- Optical spectroscopy S10
  - Additional pH titrations of **TbTb·1**: S12
  - Spectroscopy Dilution series for **TbGd·1** S14
  - Time-resolved emission profiles & Horrocks equation for **TbTb·1** S15
  - **Tb·pDO3A** model compounmd and control data S16
  - Stability of **TbTb·1** at low pH S17
  - Recomplexing the free ions after release at low pH S18
  - Control of effect of ionic strength on **TbTb·1**, addition of KCl S19
  - Control of effect of ionic stregth on **TbTb·1**, addition of K_2CO_3 .... S20
  - pH titrations of **GdGd·1**: S21
- Investigation of residual red absorption S22

Photophysical Studies S23

- Photophysical properties determined for **GdGd·1**: S23
- Quantum Yields determinations S27
- Energy Transfer investigations S29

References S33

Synthesis & Characterisation

Synthesis and Purification

Unless stated otherwise, experiments were performed at 25 °C using reagents and solvents purchased commercially and used without further purification. Anhydrous solvents were acquired by passing them through an MBraun MPSP-800 column followed by degassing with nitrogen. Triethylamine was distilled from and stored over potassium hydroxide. Deionised, microfiltered water was obtained from a Milli-Q™ Millipore machine. Merck silica gel 60 under nitrogen pressure was used for silica gel flash column chromatography. Thin layer chromatography was performed on silica-coated (60G F254) aluminium plates from Merck and aluminium oxide coated with 254 nm fluorescent indicator aluminium plates from Merck. Samples were visualized by UV-light (254 and 365 nm) and/or using permanganate stain. Solvent systems containing a mixture of solvents are reported as a ratio by volume of each solvent.

Float-A-Lyzer® G2 dialysis tubes (500, 1000 MWCO) equipped with regenerated cellulose were purchased from Spectrum and used to purify the lanthanide complexes. The dialysis tube was activated by 10 % ethanol or isopropanol solution followed by MilliQ type 1 deionised water before being used. The corresponding complexes were dissolved in water and transferred into a dialysis tube. The dialysis tube was placed in a 2.5 L-beaker filled with MilliQ type 1 deionised water. The dialysis lasts for at least two days under stirring and the deionised water was replaced with fresh deionised water more than three times during dialysis.

Characterisation Information

Mass Spectrometry

Mass spectra were carried out on a Waters BioAccord LC-MS system; flow injection analysis was performed on an ACQUITY I-Class PLUS UPLC System (Waters, Millford, MA, USA) coupled to an AQUITY RDa mass spectrometer (Waters, Milford, MA, USA) equipped with an ESI probe, in positive ion mode. The flow rate was set to 0.300 mL/min using 50 % methanol (aq) + 0.1 % formic acid eluent. Scan parameters were set as follows: analyzer mode, full scan; scan range 50-2000 m/z; scan rate, 2 Hz; cone voltage, 40 V; capillary voltage, 0.8 kV; desolvation temperature, 550 °C; and intelligent data capture, on.

NMR spectrometry

All complexes were dissolved in D_2O (0.8 mL) and added to a quartz NMR tube. To this 0.1 M NaOD was added to reach an alkaline pD (~ 10). All other pD values were reached upon sequential addition of DCl (0.1 or 0.01 M). The pD of each sample was measured with a pH Sensor InLab® NMR**.** The samples were run on either a AVD500 Bruker Avance III NMR equipped with a 11.75 T magnet (^{1}H 499.9 MHz) or a Venus400 (400 MHz) is a Bruker Avance III HD nanobay NMR equipped with a 9.4 T magnet (^{1}H 400.2 MHz). An extended sweep range of ± 750.00 ppm was used to investigate Tb(III) complexes, for Eu(III) the sweep range was extended to ± 60.00 ppm.

As the investigations were carried out in D_2O, the pD was measured. pD is related to pH using equation 5.3.[1]

$$pD = pH + 0.4$$

Synthesis of Bimetallic Complexes

TbTb·1, **EuEu·1** and **GdGd·1** were synthesised according to a previously reported procedure.[2]

EuEu·1. Yield: 35 mg (quant.); t_R= 4.33 min (CN col., 0.1% HCOOH); HR-ESI-MS obsd 1124.2194, calcd 1124.2247 [(M + H)$^+$, M = $C_{36}H_{53}N_9O_{13}Eu_2$].

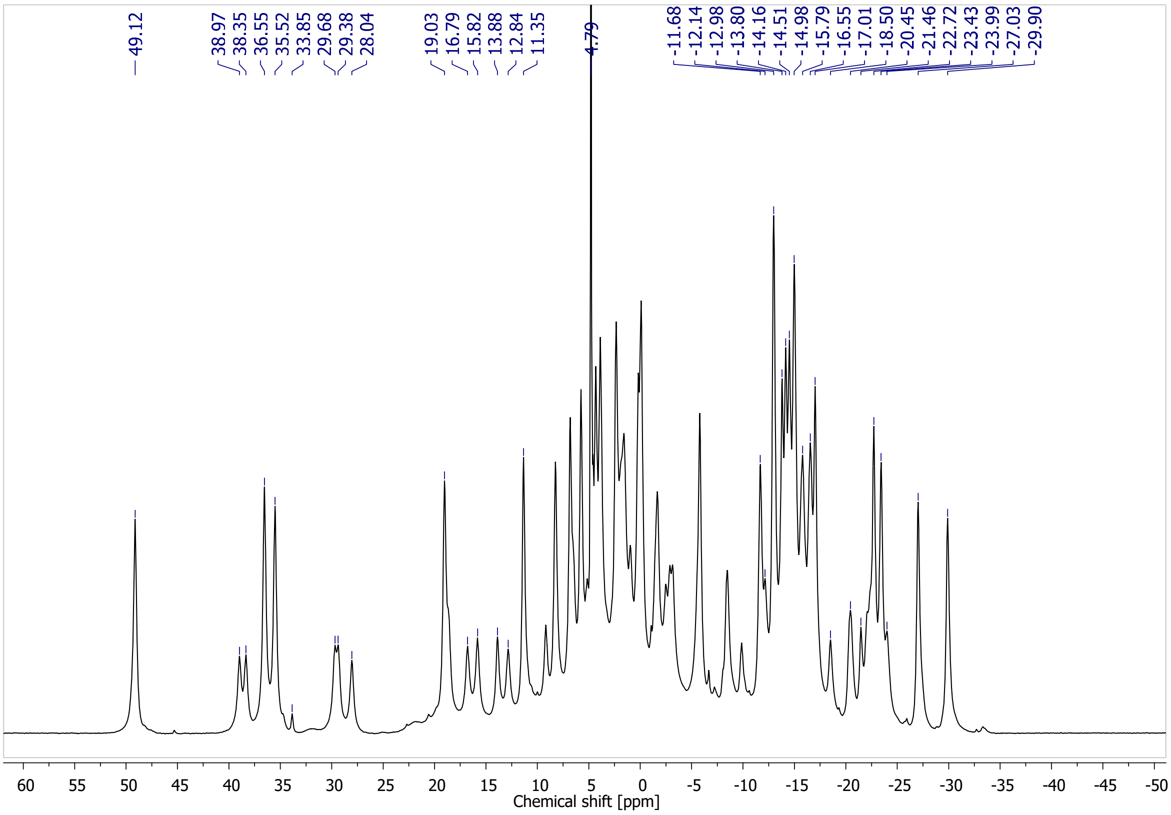

Figure S1: ^{1}H NMR spectrum of **EuEu·1** (400 MHz, D_2O).

TbTb·1. Yield: 71 mg (quant.); t_R= 4.24 min (CN col., 0.1% HCOOH); HR-ESI-MS obsd 1138.2320, calcd 1138.2343 [(M + H)$^+$, M = $C_{36}H_{53}N_9O_{13}Tb_2$].

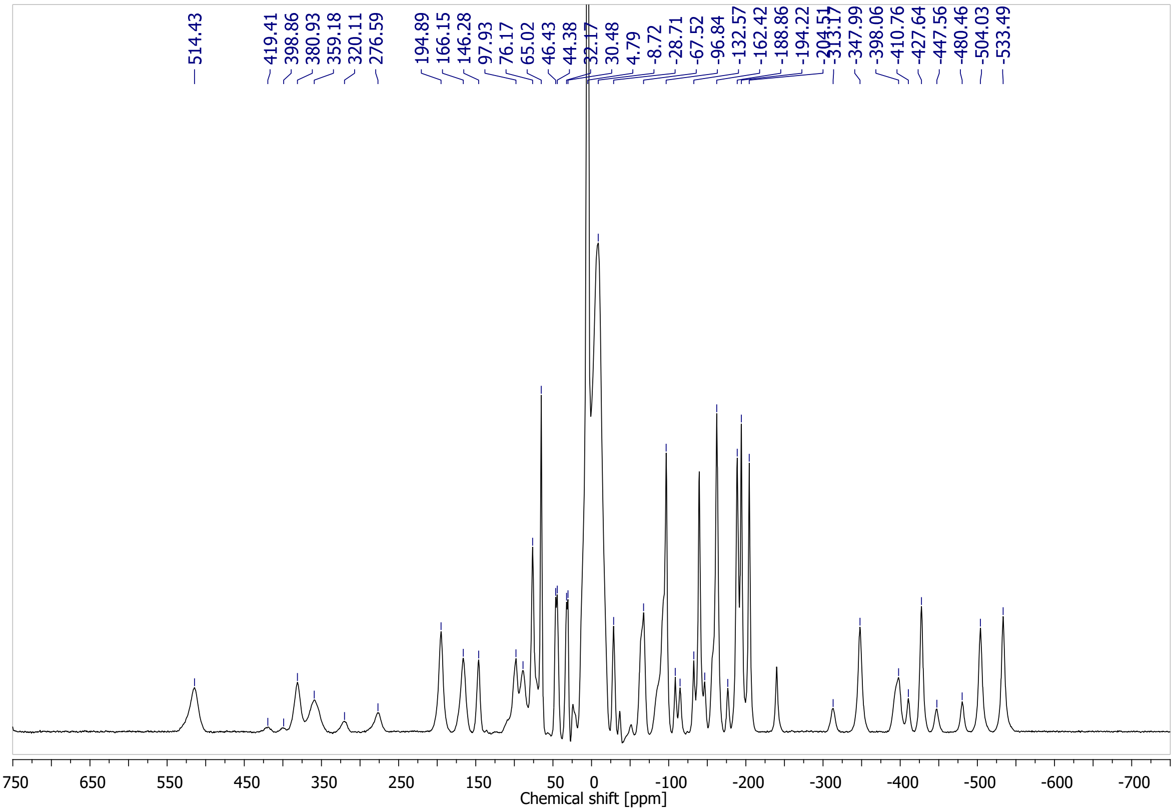

Figure S2: ^{1}H NMR spectrum of **TbTb'1** (500 MHz, D_2O).

GdGd'1 . Yield: 29 mg (quant.); t_R= 4.28 min (CN col., 0.1% HCOOH); HR-ESI-MS obsd 1136.2304, calcd 1136.2318 $[(M + H)^+$, $M = C_{36}H_{53}N_9O_{13}Gd_2]$.

Synthesis of Heterobimetallic Complexes

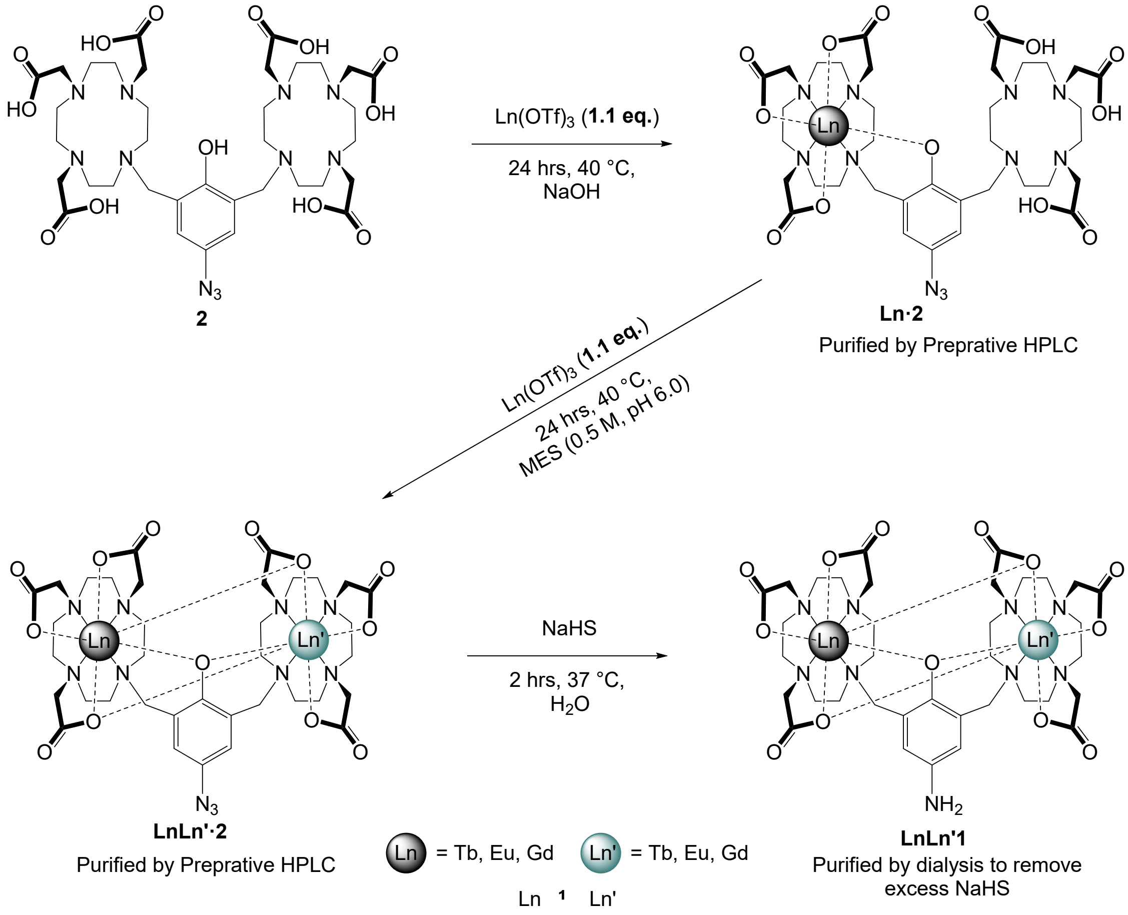

Scheme S1: Synthetic procedure for the synthesis of Heterobimetallic Ln(III) complexes. L_2 was made according to a previously reported procedure.[2a]

Preparation of the monometallic complexes

The appropriate ligand (1.0 eqv.) and the corresponding Ln-triflate (1.1 eqv.) were mixed and then dissolved in H_2O (c_{ligand} = 0.05M). The reaction mixture was heated to 40 °C and after 1 hour of stirring 1M $NaOH_{(aq)}$ solution (3.5 eqv) was added, and the complexation was allowed to run for 18 hours. At this point, two more portions of 1M $NaOH_{(aq)}$ was added with 1 hour time difference (2.5 + 1.0 eqv). The resulting mixture was filtered through a membrane filter (Nylon, 0.45 µm pore size) and the filtrate was directly injected onto and purified by preparative HPLC. As eluents, 25 mM NH_4HCO_3 aqueous solution and MeCN were used. Gradient elution (11 min method: 0-5 min, 5% MeCN (iso); 5-7 min, 5-95% MeCN (grad); 7-8 min, 95% MeCN (iso), 8-10 min 95-5% MeCN (grad), 10-11 min, 5% MeCN (iso); flow rate: 15

mL/min; Column: Agilent 5 Prep C18 P/N 446905-702, 50 × 21.2 mm) resulted both mono- and binuclear complexes, which could be separated by preparative HPLC.

Tb·2. Yield: 221 mg (16%); t_R= 11.84 min (CN col., 0.1% HCOOH); ESI-MS obsd 1008.10, calcd 1008.32 (M + H)$^+$; HR-ESI-MS obsd 1008.3203, calcd 1008.3229 [(M + H)$^+$, M = $C_{36}H_{54}N_{11}O_{13}Tb$].

Preparation of the heterobinuclear complexes

To a stirred solution of the appropriate mononuclear complex (1.0 eqv) in 0.5M MES buffered aqueous solution (at pH 6.0) was added the corresponding Ln-triflate (1.1 eqv). The resulting mixture was allowed to stir at 40°C for 18 hours. The mixture was filtered through a membrane filter (Nylon, 0.45 µm pore size) and the filtered solution was directly injected onto the preparative HPLC. As eluents MilliQ type 1 deionised water (without any additives) and MeCN were used. Gradient elution (11 min method: 0-5 min, 5% MeCN (iso); 5-7 min, 5-95% MeCN (grad); 7-8 min, 95% MeCN (iso), 8-10 min 95-5% MeCN (grad), 10-11 min, 5% MeCN (iso); flow rate: 15 mL/min, Column: Agilent 5 Prep C18 P/N 446905-702, 50 × 21.2 mm) resulted the pure heterobinuclear complexes.

TbEu·2. Yield: 134 mg (71%); t_R= 12.86 min (CN col., 0.1% CH_3COOH); ESI-MS obsd 1157.71, calcd 1158.22 (M + H)$^+$; HR-ESI-MS obsd 1158.2182, calcd 1158.2207 [(M + H)$^+$, M = $C_{36}H_{51}TbEuN_{11}O_{13}$].

TbGd·2. Yield: 56 mg (60%); t_R= 12.58 min (CN col., 0.1% HCOOH); ESI-MS obsd 1163.88, calcd 1163.22 (M + H)$^+$; HR-ESI-MS obsd 1163.2206, calcd 1163.2235 [(M + H)$^+$, M = $C_{36}H_{51}N_{11}O_{13}TbGd$].

Example of reduction of Heterobimetallic complexes

TbEu·2 (0.0236 mmol, 1.00 eqv) was dissolved in D_2O (280 µL) followed by the addition of NaHS·xH_2O (141 µL, 0.141 mmol (1 M solution in D_2O), 6.00 eqv) and the reaction was let to stir at 37 °C for 2 hours. In the first 30 mins intense effervescence was observed. The reaction mixture was centrifuged and the supernatant was decanted and purfied by dialysis. The solvent was removed under continuous N_2 flow at 50 °C to result the reduced complex, **TbEu·1**, as a dark purple solid.

TbEu·1. Yield: 30 mg (quant.); t_R= 4.66 min (CN col., 0.1% CH_3COOH); ESI-MS obsd 1131.85, calcd 1131.22 (M + H)$^+$; HR-ESI-MS obsd 1154.2112, calcd 1154.2121 [(M + Na)$^+$, M = $C_{36}H_{53}N_9O_{13}EuTb$].

Investigation of Speciation

1H NMR spectroscopy

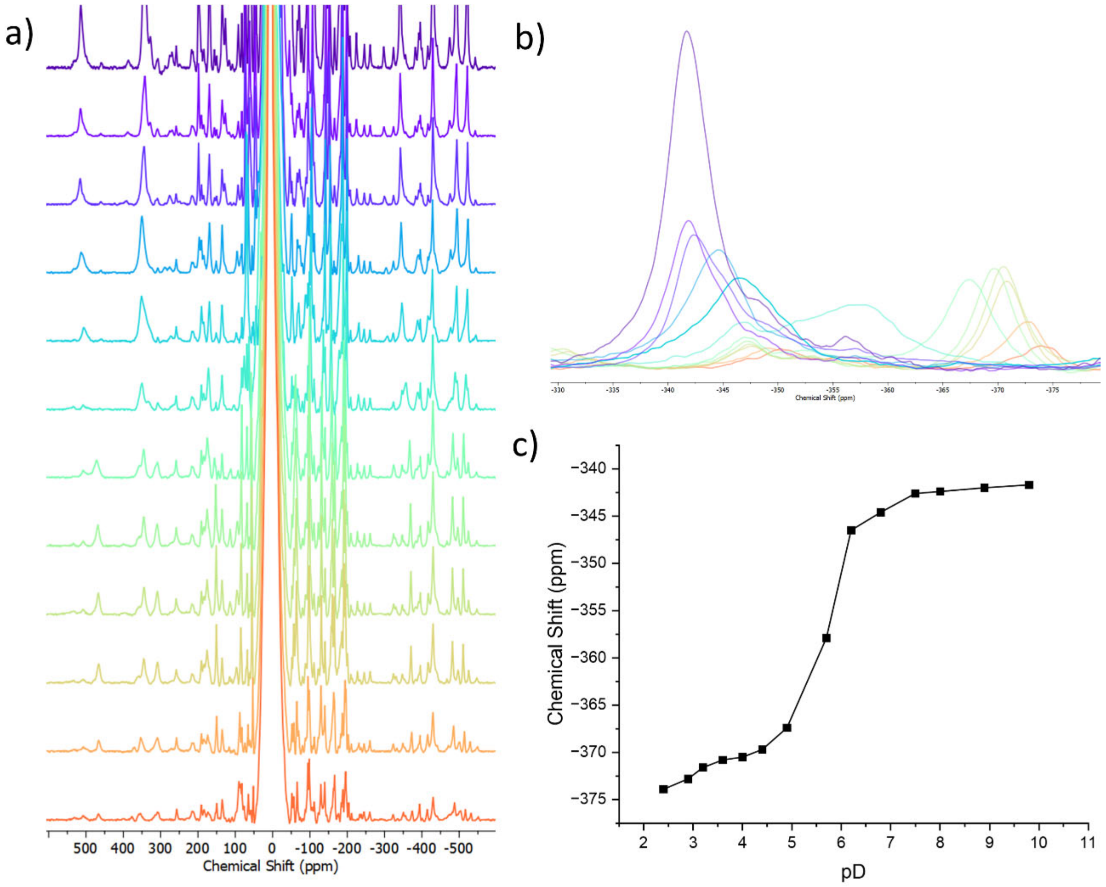

Figure S3: ^{1}H NMR investigations into **TbTb·1** in D_2O, a) Stacked ^{1}H NMR (499.9 MHz) spectra in D_2O at varying pD's, b) Superimposed spectra, detailing the changes observed in the proton environment between -340--375 ppm, upon changing pD, c) chemical shift vs pD of proton environment between -340--375 ppm.

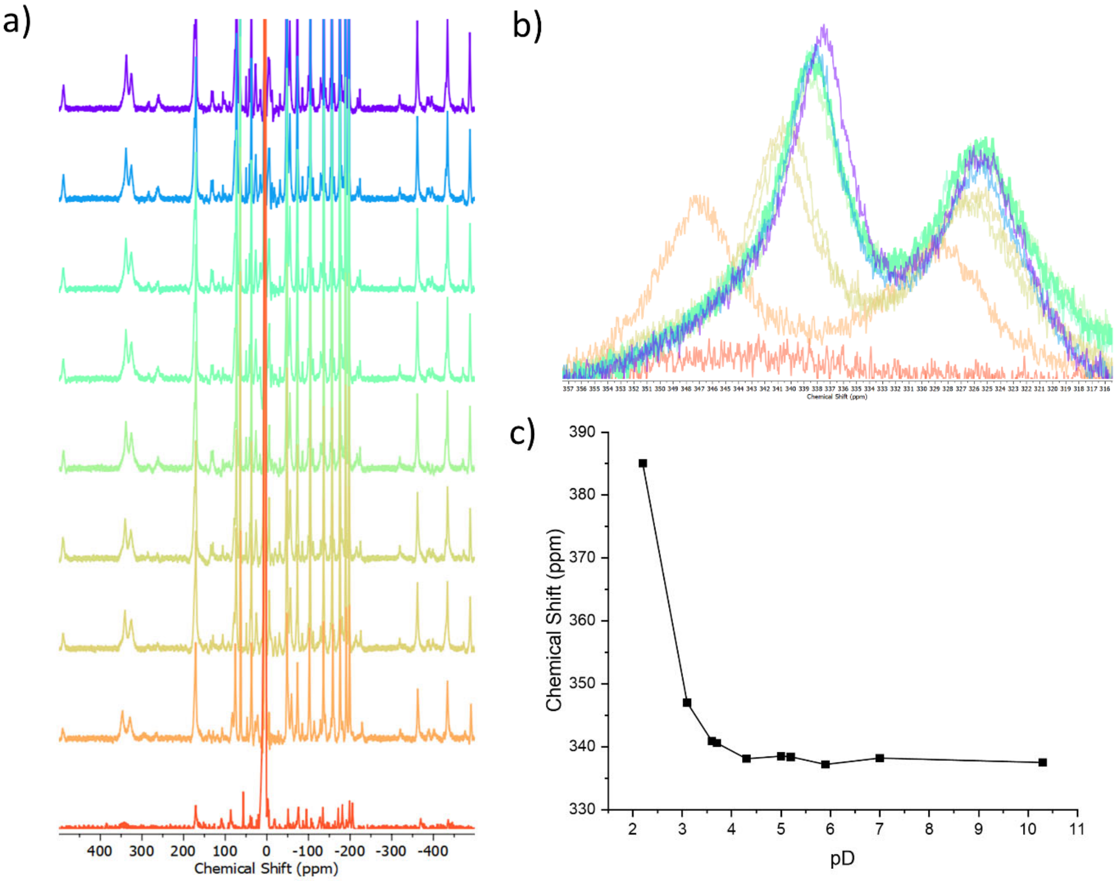

Figure S4: ^1H NMR investigations into **TbTb·1** in D_2O, a) Stacked ^1H NMR (499.9 MHz) spectra in D_2O at varying pD's, b) Superimposed spectra, detailing the changes observed in the proton environment between 330-390 ppm, upon changing pD, c) chemical shift vs pD of proton environment between 330-390 ppm.

Optical spectroscopy

Experimental details.

All measurements were carried out in a 10 mm cuvettes from Starna Scientific.
Absorption measurements were performed on a Cary5000 double-beam spectrophotometer from Agilent Technologies. Absorption was measured on an absolute scale with water as a reference (transmission = 100%) and a blocked beam (transmission = 0%). Slits were kept at 2 nm.

Steady-state and time-resolved emission spectra were measured on a QuantaMaster8075 from Horiba Scientific using a Xenon-lamp as excitation source.
Terbium(III) and europium(III) centered steady state emission were collected with 0.6 nm emission slits and 8 nm excitation slits. The step size was 0.3 nm with 0.2 s integration time. Organic chromophore centered steady state emission were collected with 2 nm emission slits and 5 nm excitation slits. The step size was 1 nm with 0.1 s integration time.
Steady state excitation spectra were collected with 5 nm emission slits and 2 nm excitation slits. The step size was 1 nm with 0.1 s integration time.
Time resolved emission spectra were collected with 5 nm emission slits and 8 nm excitation slits.
Luminescence decay measurements were measured with 8 nm emission slits and 8 nm excitation slits. The data range is from 200 µs to 20,000 µ, the lamp frequency was 50 ms. Each decay is measured with 2000 shots and no binning has been done.

Horrocks Equation

The hydration number q, i.e. the amount of water molecules bound in the first coordination sphere of Tb(III) in a complex can be estimated from the luminescent lifetimes using the Horrocks' equation.[3] The equation is defined in equation S1.

$$q = \mathrm{A}[\tau_{H2O}^{-1} - \tau_{D2O}^{-1}] \qquad \text{Eq. S1}$$

Where, τ_{H2O} is the lifetime in water and τ_{D2O} is the lifetime in deuterated water. A is an empirical parameter, which for Tb(III) is 4.2.[3b]

Table S1. Photophysical information of the four species of LnLnL. The excitation ($\lambda_{exc,max}$) and absorbance ($\lambda_{abs,max}$) maxima is reported for the TbTbL complex. The fluorescence maxima ($\lambda_{fluor,max,L}$), phosphorescence maxima ($\lambda_{fluor,max,L}$), and phosphorescence lifetime ($\tau_{L,phos}$) of the Ligand is measured with GdGdL. The Tb(III) emission lifetimes (τ_{H2O} and τ_{D2O}), the q-value (q) and the quantum yield of Tb(III) (QY_{Tb}) is measured with TbTbL.

Species	pH	$\lambda_{exc,max}$ (nm)	$\lambda_{abs,max}$ (nm)	S1 (cm^{-1})	T1 (cm^{-1})	T1,C (cm^{-1})	$\lambda_{fluor,max,L}$ (nm)	$\lambda_{phos,max,L}$ (nm)	$\tau_{L,phos}$ (ms)	τ_{H2O} (ms)	τ_{D2O} (ms)	q	QY_{Tb} %
TbTb Species 1	7	314	311	29420	-	-	-	-	-	2.71	2.83	0.07	43.5
TbTb Species 2	3.5	285	285	32840	-	-	-	-	-	2.80	2.86	0.03	11.6
TbTb Species 3	2.4	302	303	30780	-	-	-	-	-	1.90	2.56	0.57	~6
TbTb Species 4	0.7	-	284	-	-	-	-	-	-	-	-	-	0.0
GdGd Species 1	9	-	311	29410	25380	25960	376	442	4.7	-	-	-	-
GdGd Species 2	3.2	-	286	33110	28570	25990	~310	385	0.6	-	-	-	-
TbGd Species 1	7	315	311	29370	-	-	-	-	-	2.52	-	-	46.9
TbEu Species 1	7	315	311	29380	-	-	-	-	-	1.73	-	-	0.261

Additional pH titrations of **TbTb·1**:

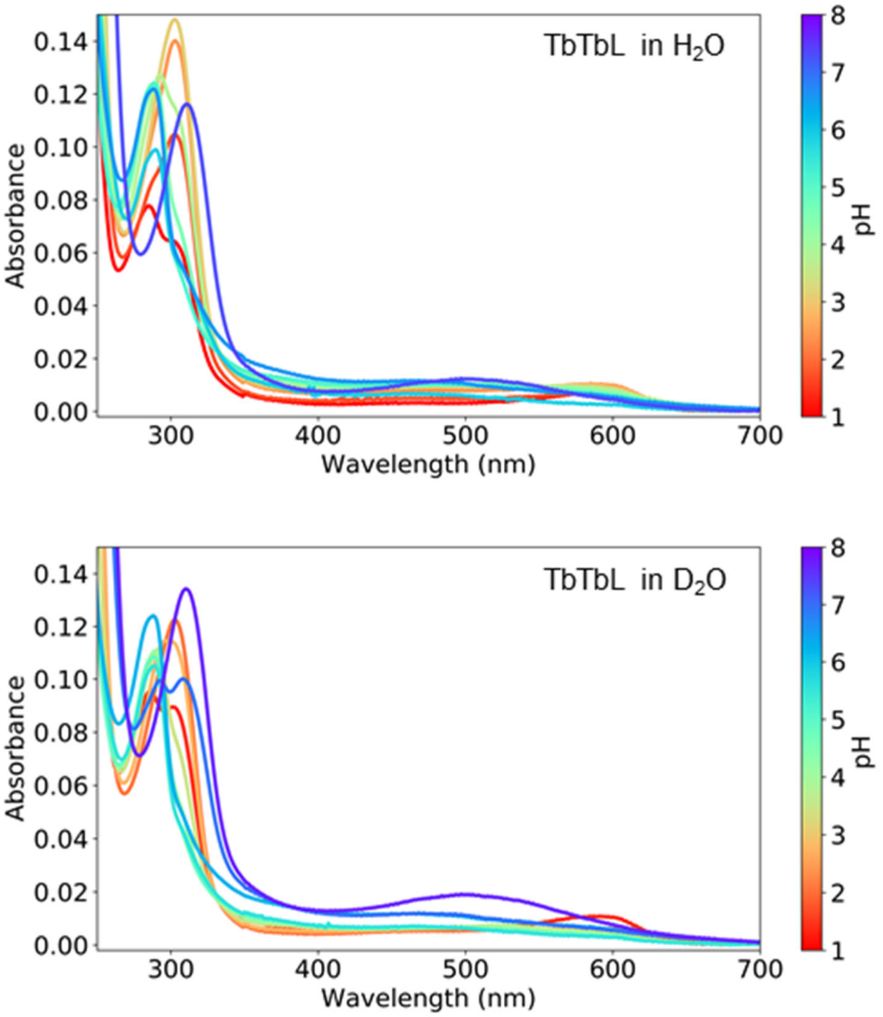

Figure S5: Absorption spectra of **TbTb·1** in H_2O (top) and D_2O (bottom) at pH values from 8 to 1.

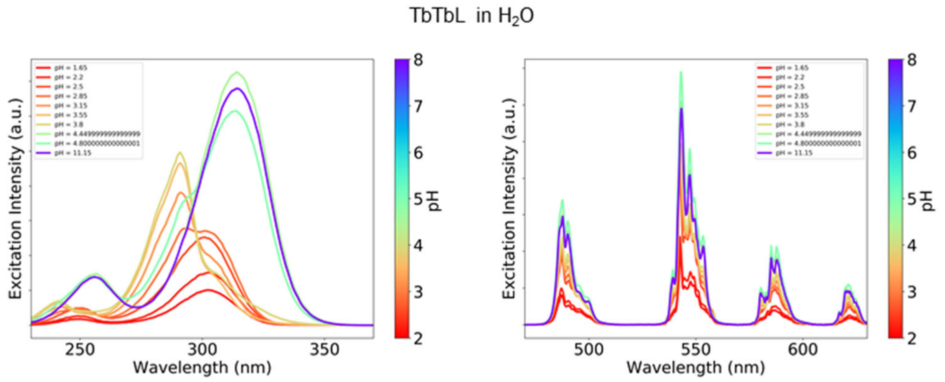

Figure S6**:** Excitation spectra, em = 545 nm, (left) and emission spectra, exc = 295 nm, (left) of **TbTb·1** in H_2O at pH (top) and D_2O (bottom) at pH values from 8 to 2.

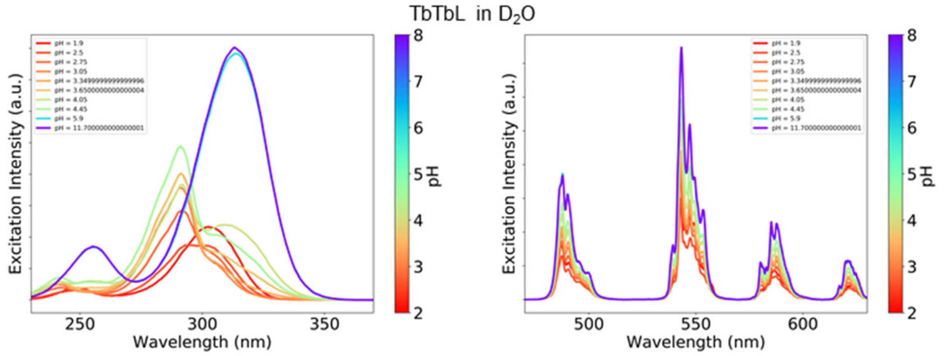

Figure S7: Excitation spectra, em = 545 nm, (left) and emission spectra, exc = 295 nm, (left) of **TbTb·1** in D_2O at pH (top) and D_2O (bottom) at pH values from 8 to 2.

Spectroscopy Dilution series for TbGd·1

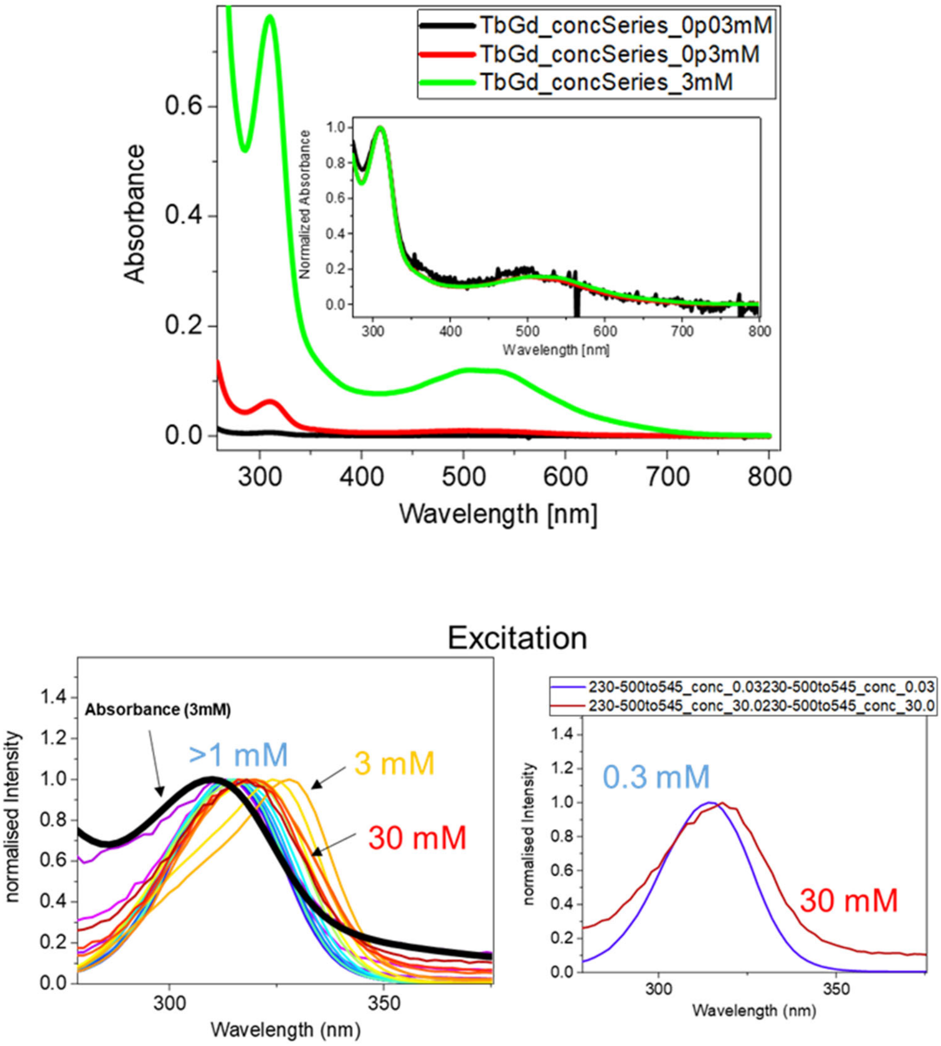

Figure S8: Top: Absorbance of **TbGd·1** at different concentrations at pH = 7. Inlet shows normalized spectra, revealing no spectral differences. Bottom: Normalized excitation spectra at varying concentrations.

Time-resolved emission profiles & Horrocks equation for **TbTb·1**

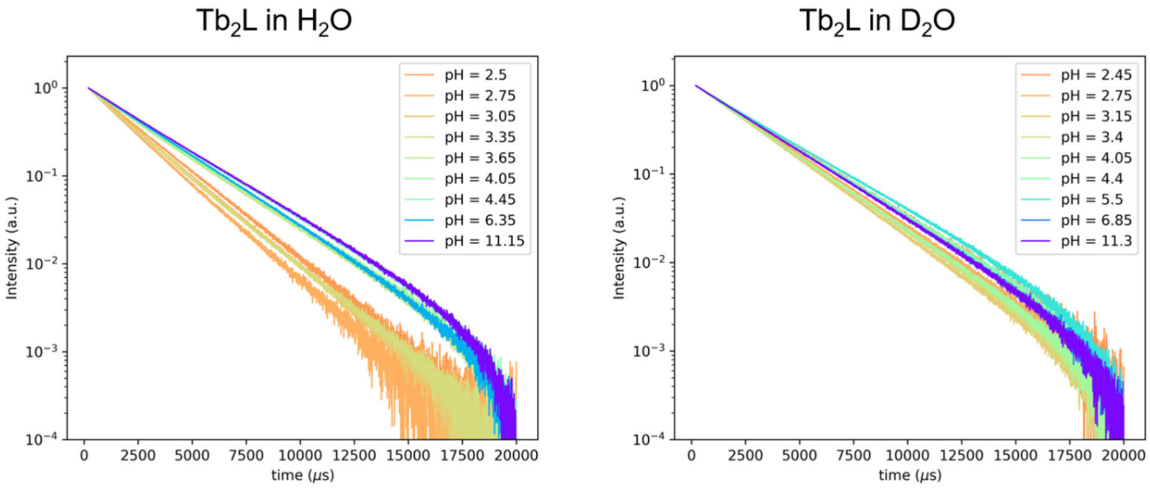

Figure S9: Lifetimes of **TbTb·1** at varying pH values in H_2O and measured pH values in D_2O

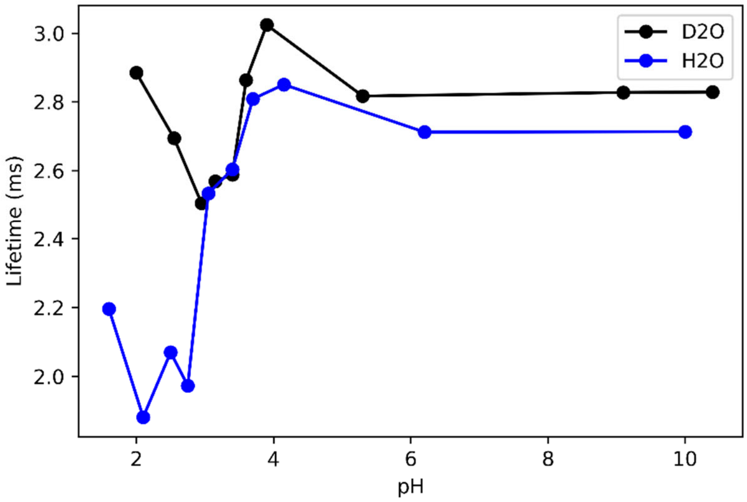

Figure S10: Fitted lifetimes of **TbTb·1** in H_2O and D_2O as a function of pH. pH values for D_2O is adjusted by adding 0.4 to the measured value.

Tb·pDO3A model compounmd and control data

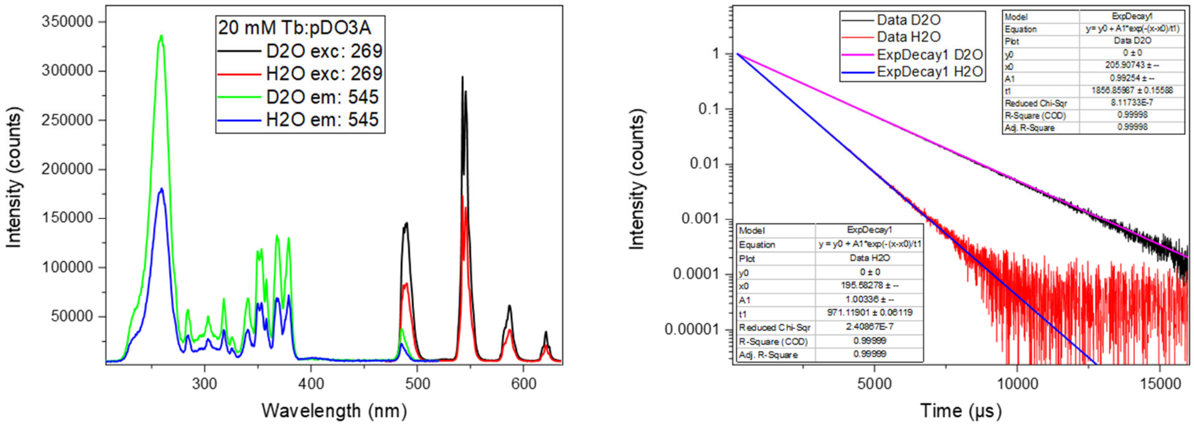

Figure S11: Left: Excitation spectra (em: 545 nm) and emission spectra (exc: 269 nm) of 20 mM **Tb·pDO3A** in H_2O and D_2O at pH = 5. Right: Lifetimes that have been fitted of **Tb·pDO3A** in H_2O and D_2O. The difference between these lifetimes corresponds to a q-value of 2.

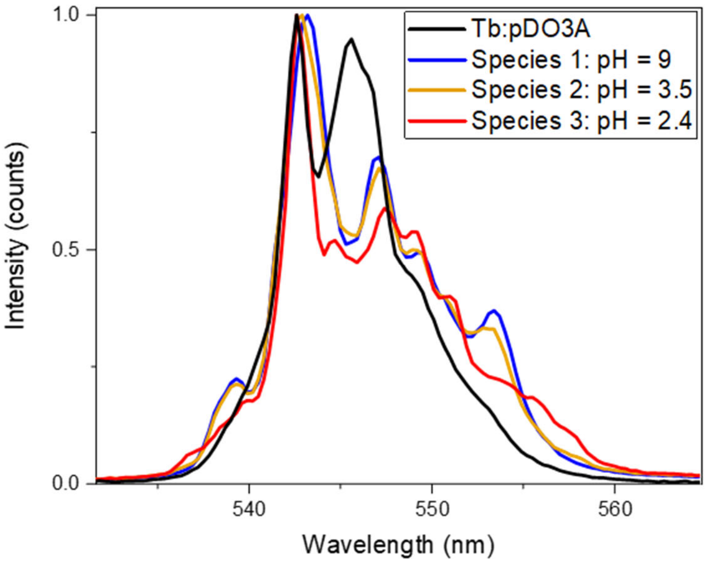

Figure S12: Normalized emission spectra of the $^5D_4$ to $^7F_5$ band of Tb(III) in **TbTb·1** at pH = 9, 3.5, and 2.4 and for the **Tb·pDO3A** complex at pH = 5.

Stability of TbTb·1 at low pH

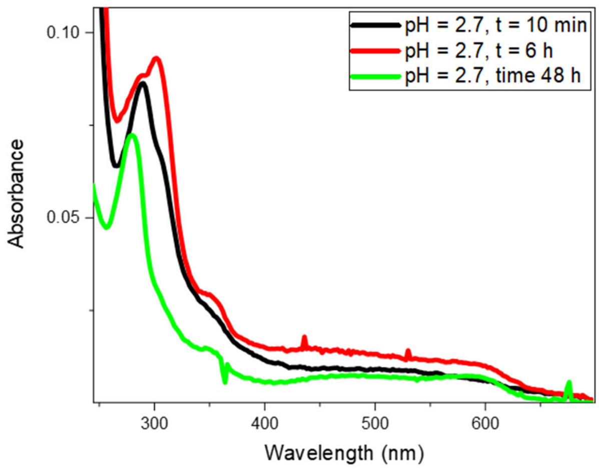

Figure S13: Absorbance spectra of the **TbTb·1** at 10 min, 6 h, and 48 h after the pH is decreased to pH = 2.7.

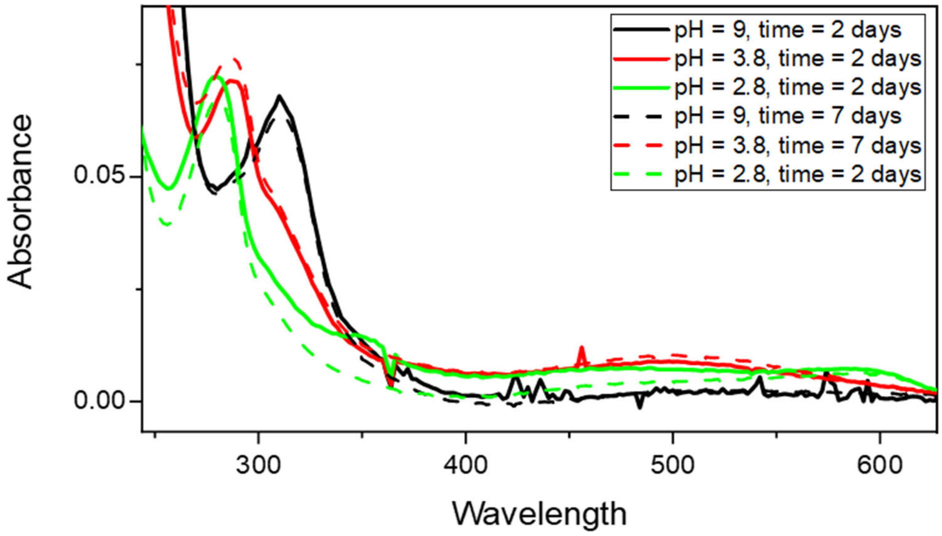

Figure S14: Absorbance spectra of **TbTb·1** 2 days and 7 days after the pH has been adjusted to pH = 9, 3.8, and 2.8.

Recomplexing the free ions after release at low pH

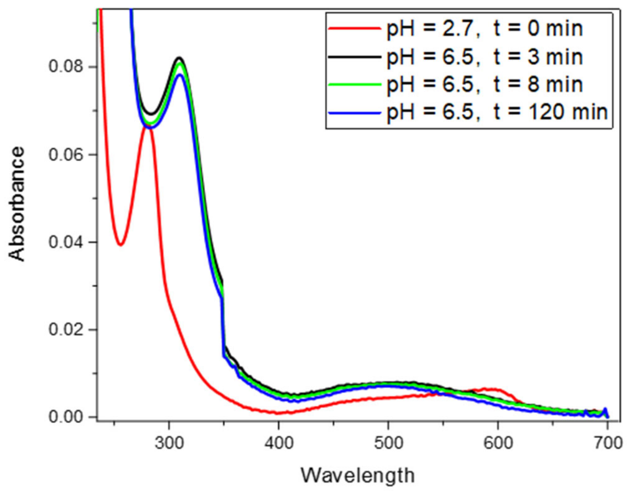

Figure S15: In-situ Absorption spectra of the **TbTb·1** as the pH is increased from pH = 2.7 to 6.5. Timestamps are shown for 0 min at pH 2.7 and 3, 8, and 120 min at pH 6.5. Note that the sudden drop in absorbance at 350 nm is an artefact due to a lamp change. The absorbance above 350 nm is consequently ca. 0.8 too high for the three samples where this artefact is observed.

Control of effect of ionic strength on TbTb·1, addition of KCl

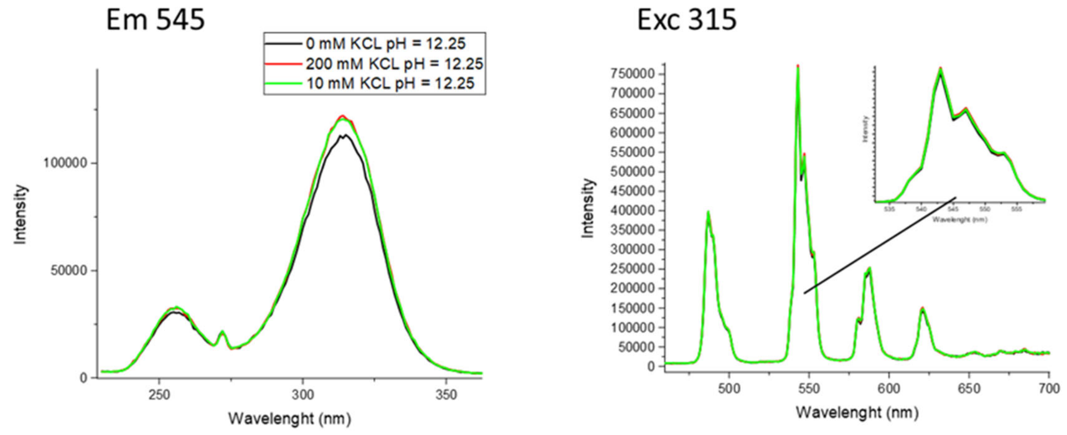

Figure S16: Spectra of **TbTb·1** at pH = 12.25 with 0, 10, and 200 mM added KCL. Left: Excitation spectra recording emission at 545 nm. Right: Emission spectra following excitation at 315 nm. Insert shows a zoom in on the 545 nm band with no spectral changes.

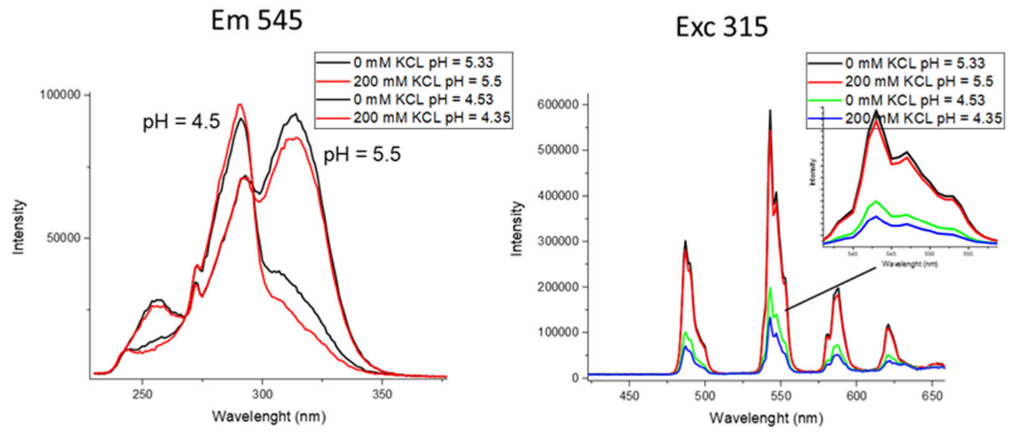

Figure S17: Spectra of **TbTb·1** at pH = 4.5 and pH = 5.5 with 0 and 200 mM added KCL. Left: Excitation spectra recording emission at 545 nm. Right: Emission spectra following excitation at 315 nm. Insert shows a zoom in on the 545 nm band with no spectral changes.

Control of effect of ionic stregth on **TbTb·1**, addition of K_2CO_3

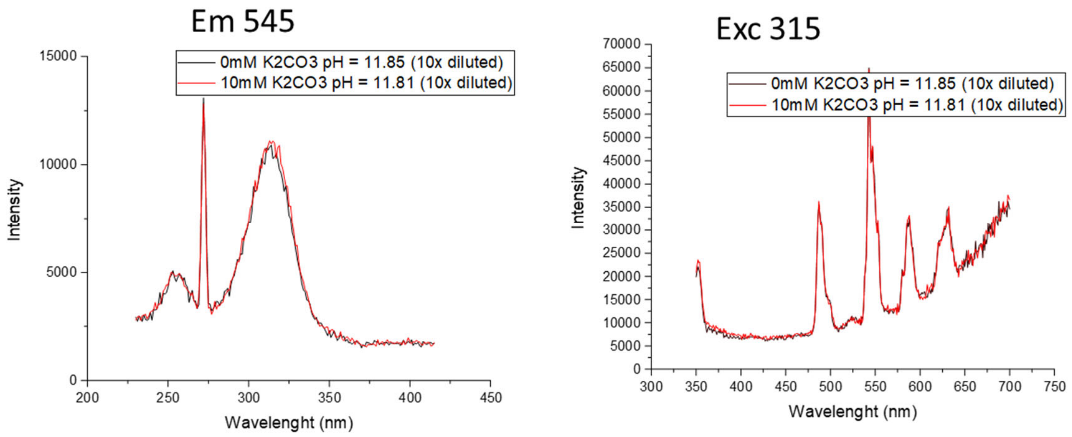

Figure S18: Spectra of **TbTb·1** at pH = 11.85 with 0 and 10 mM added K_2CO_3. Left: Excitation spectra recording emission at 545 nm. Right: Emission spectra following excitation at 315 nm. No spectral changes are found

pH titrations of **GdGd·1**:

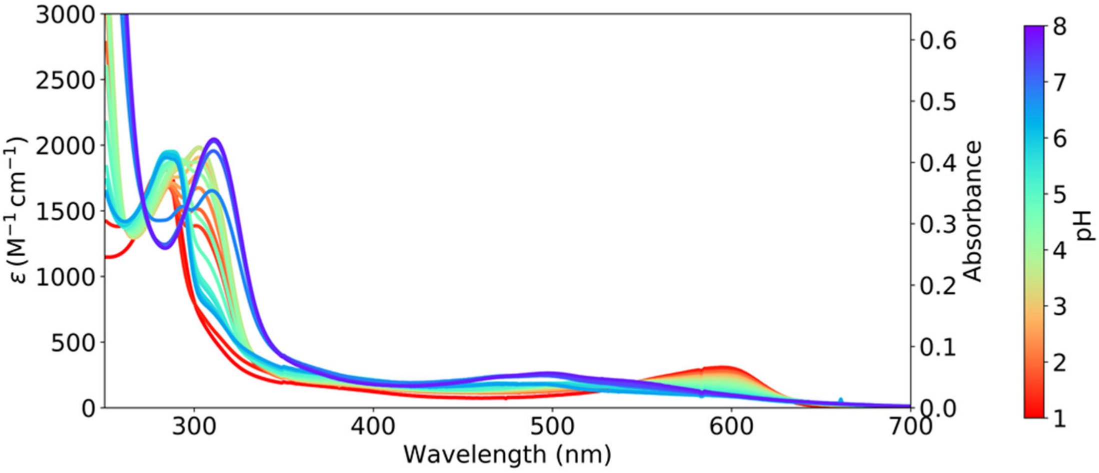

Figure S19: Absorbance and the calculated extinction coefficient of **GdGd·1** at varying pH values from 8 to 1.

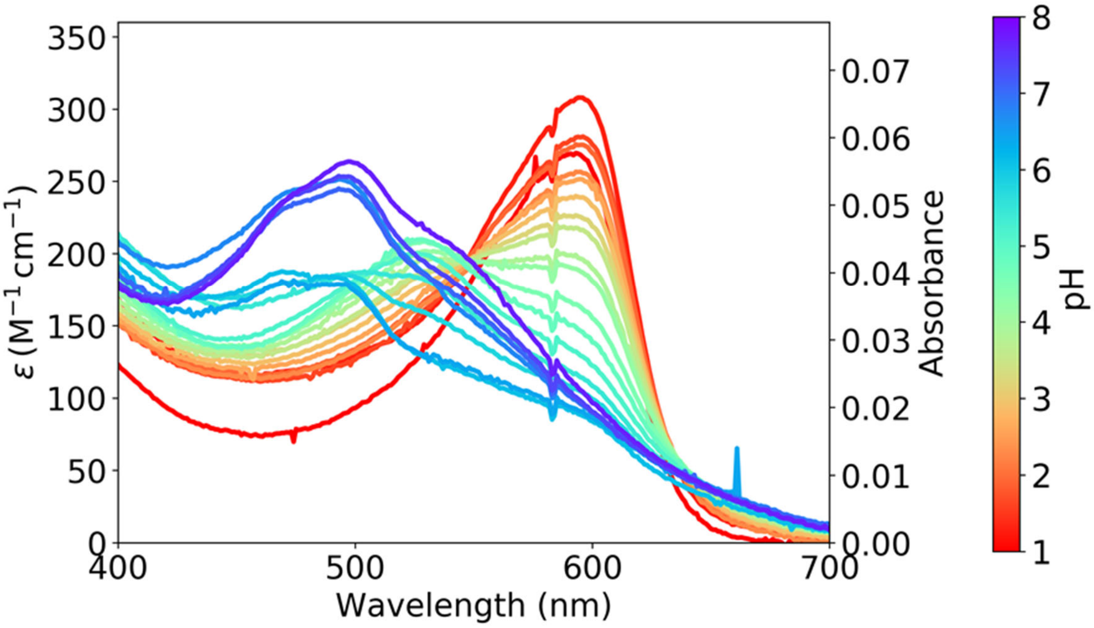

Figure S20: Absorbance and the calculated extinction coefficient **GdGd·1** at varying pH values from 8 to 1. Zoomed in on the 400 to 700 nm range where residual absorption from partial reduction of the azide is seen. Note that the repeating dip in absorbance at ca. 580 nm is an artefact of the recorded background.

Investigation of residual red absorption

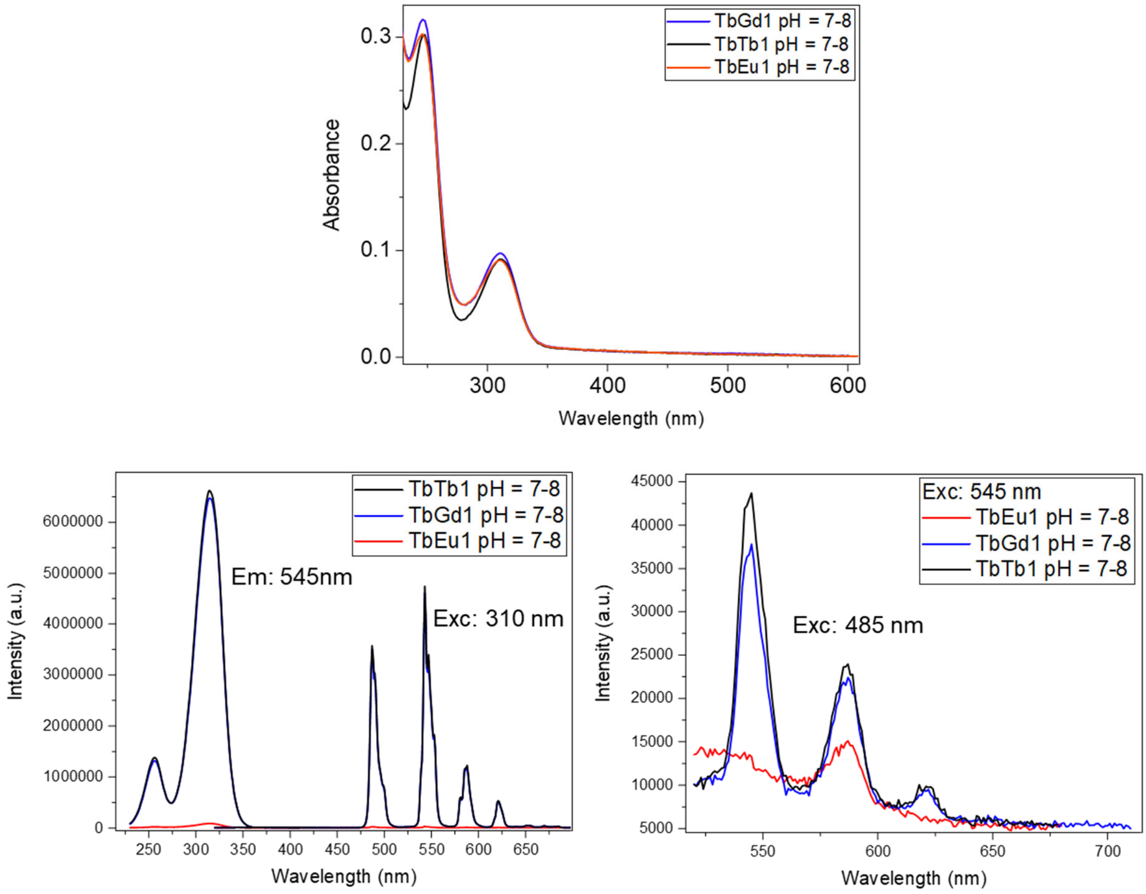

Figure S21: Absorption, emission **and e**xcitation spectra of a preparation of **TbTb·1**, **TbGd·1**, and **TbEu·1** with excess reductant. Note that the red absorption has been fully removed.

Photophysical Studies

Photophysical properties determined for **GdGd·1**:

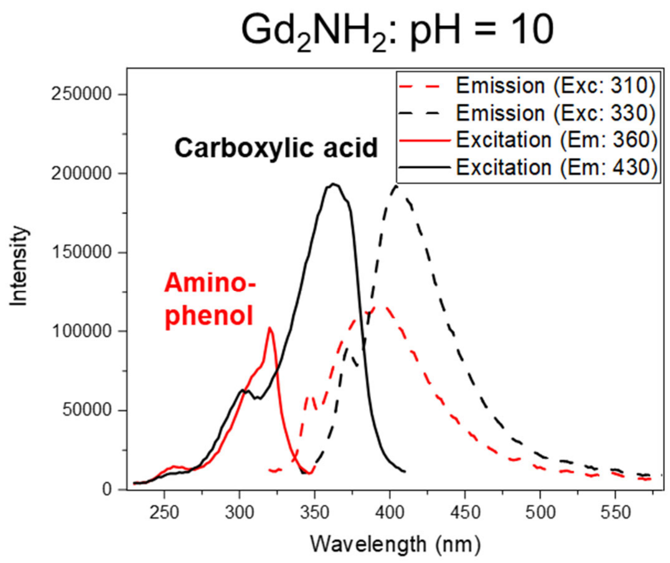

Figure S22: Excitation and emission spectra **GdGd·1** at pH = 10. Excitation is showed for recorded emission at 360 and 430 nm emission and Emission is showed following excitation at 310 and 330 nm. The excitation spectra recorded from 360 emission matches the absorbance of the compound, resultantly; this is believed to be corresponding to the amino-phenol. The other component is assigned to be the carboxylic acid arms, which displays phosphorescence.

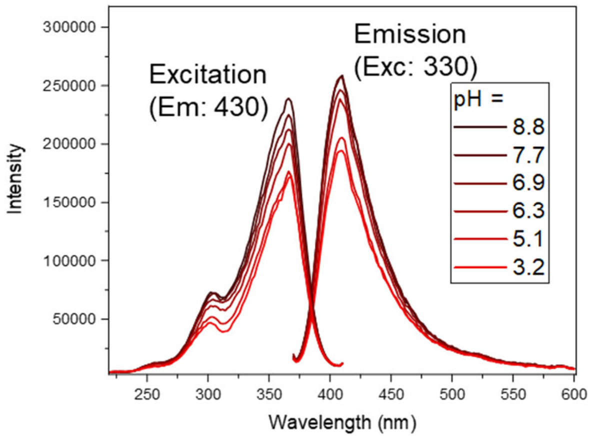

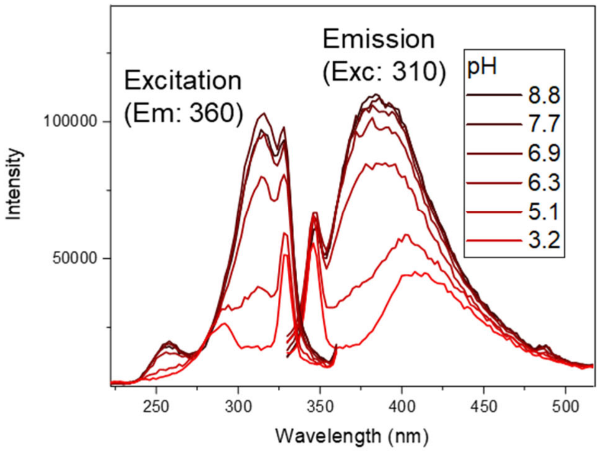

Figure S23: Left: Excitation (em: 430 nm) and emission (exc: 330 nm) spectra **GdGd·1** at varying pH. The change in peak positions are insignificant, which would be expected for the carboxylic acid arms in DO3A. Right: Excitation (em: 360 nm) and emission (exc: 430 nm) spectra **GdGd·1** at varying pH. The change in peak positions at pH = 5 occurs similarly, with a slight blue-shift as the L:TbTb variant and the changes can be assigned to be equivalent. The emission is near-zero however and the emission at pH 3.2 is dominated by the carboxylic acid phosphorescence. Furthermore, the large peaks at excitation ~330 nm and emission ~345 nm is assigned to be Raman scattering.

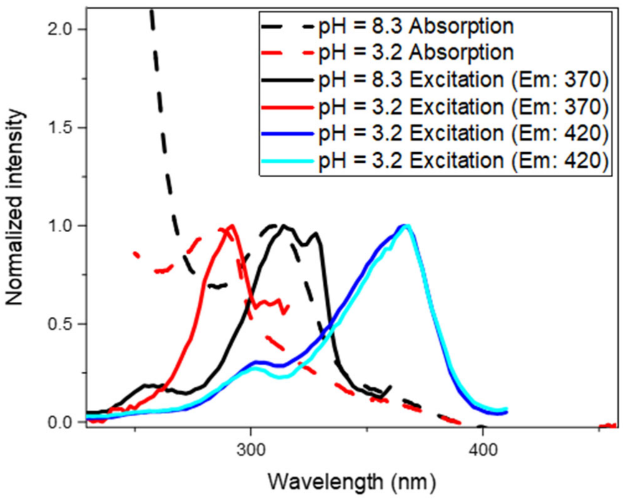

Figure S24: Combined and overlaid absorption and excitation spectral species as found for the **GdGd·1** compound at pH value 8.3 and 3.2. The spectra assigned to be of the amino phenol is found to overlap well with the absorbance spectra and the spectra assigned as the carboxylic acid arms are identical at the two pH.

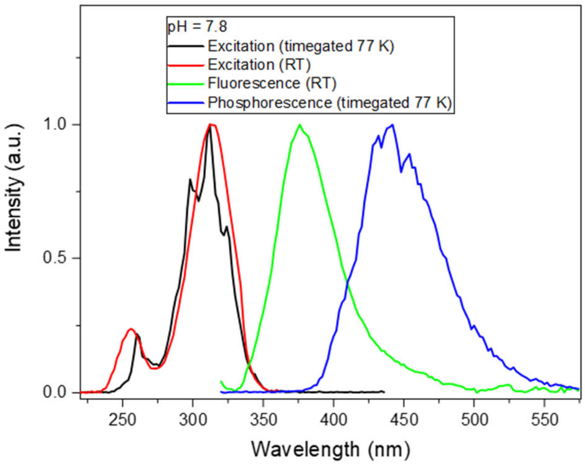

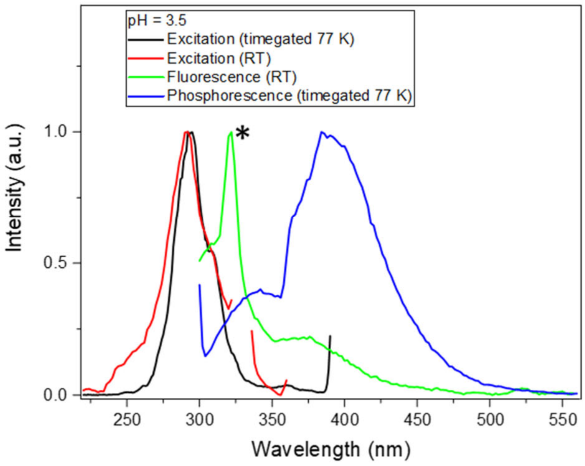

Figure S25: Steady state excitation and emission spectra of **GdGd·1** measured at room temperature overlaid with time-gated measurements at 77 K at pH = 7.8 (left) and pH = 3.5 (right). The phosphorescence is measured between 200 µs and 1000 µs after excitation.

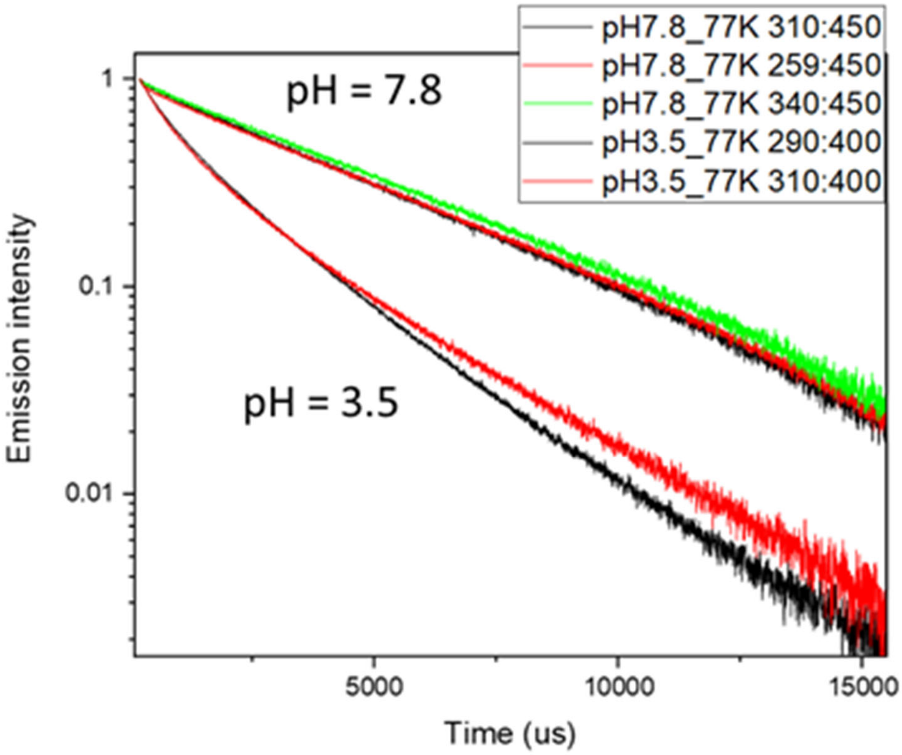

Figure S26: Lifetimes of **GdGd·1** measured at pH 7.8 and 3.5 at different wavelengths of excitation. pH = 3.5 is measured from excitation at 290 and 310 nm and with emission at 400 nm. pH = 7.8 is measured from excitation at 259, 310, and 340 nm and with emission at 450 nm. The pH = 7.8 is found to be invariant with excitation wavelength, whereas the pH 3.5 has two components. The long component represented more from excitation at 310 nm.

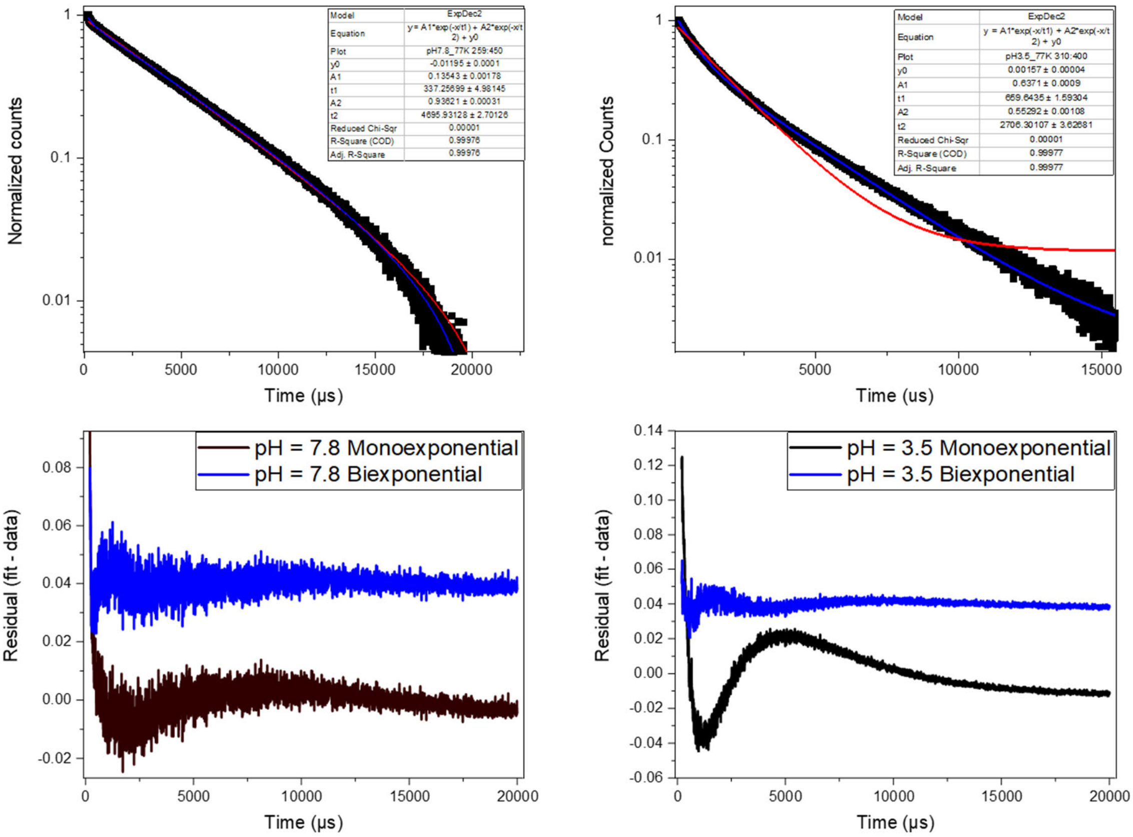

Figure S27. Top: Fits of the lifetimes of **GdGd·1** measured at pH 7.8 and 3.5. Bottom: Residual plots for both mono exponential and bi exponential fits.

Quantum Yields determinations

The luminescence quantum yield was determined using the IUPAC recommended 5-point dilution method with quinine sulphate in 0.5 M H_2SO_4 in water as a reference standard. Quantum yield of this standard is accepted as 0.546.[4] The quantum yields Φ_{lum} where measured with Eq. S2.

$$\Phi_{\mathrm{lum}} = \Phi_{\mathrm{ref}} \frac{\int I_X^{Em}}{\mathrm{A_X}} \frac{\mathrm{A_{ref}}}{\int I_{ref}^{Em}} \qquad \text{Eq. S2}$$

Where Φ_{lum} and Φ_{ref} are the quantum yields of the sample and reference respectively. $\int I_X^{Em}$ and $\int I_{ref}^{Em}$ are the total integrated emission intensity of the sample and reference measured with the same excitation wavelength respectively. $\mathrm{A_X}$ and $\mathrm{A_{ref}}$ are the absorbance at the excitation wavelength for the sample and reference respectively. The refractive indexes of both solvents are the same as both measurements are performed with water as the solvent. The data and the processing of Eq. S2 are shown in Figure S28.

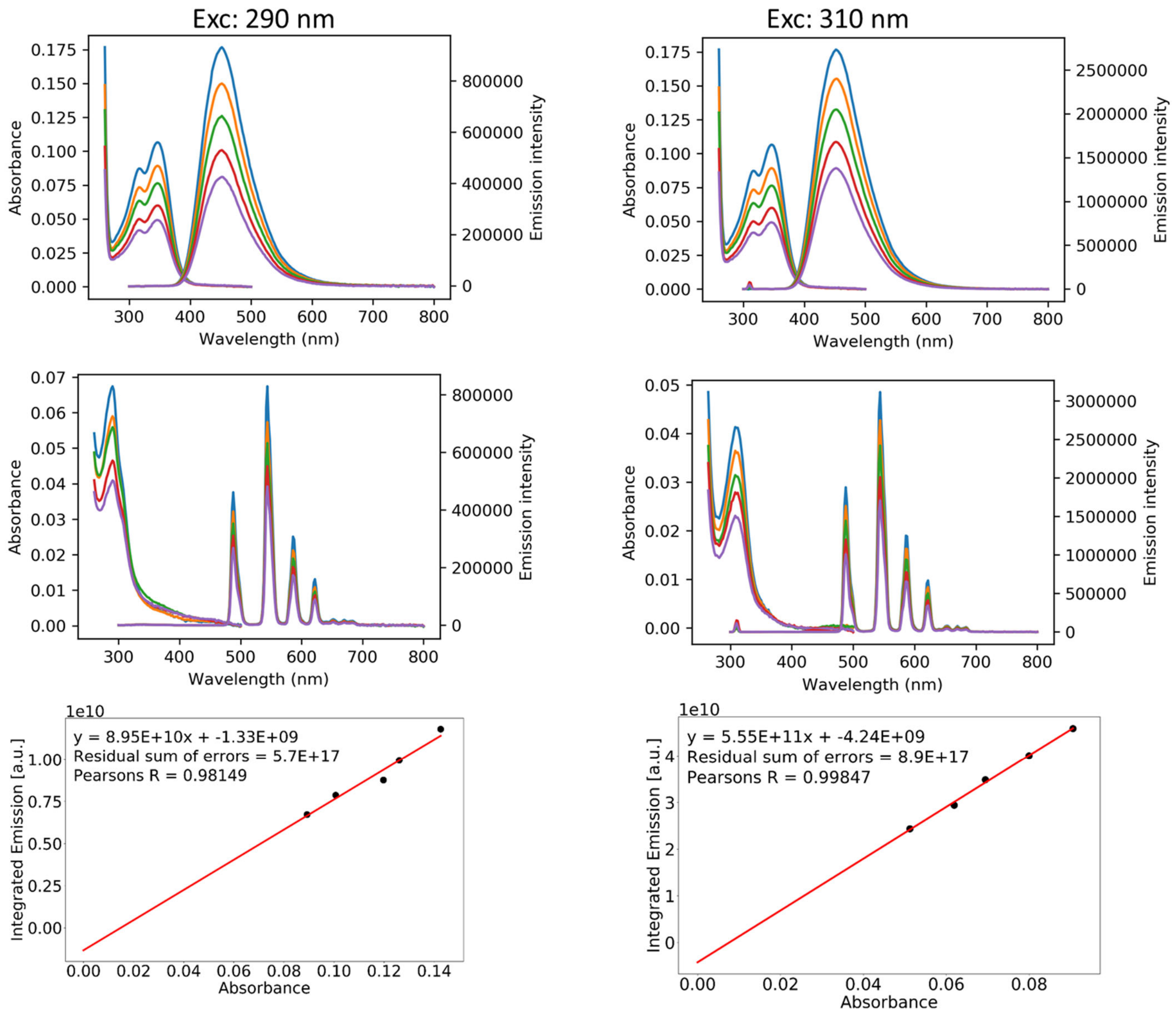

Figure S28: 5-point dilution series for determination of quantum yields. Absorbance and emission of quinine sulphate reference (top) and of **TbTb·1** (middle) and the corresponding integrated emission as a function of absorbance (bottom). Left: shows excitation at 290 nm where **TbTb·1** is measured at pH = 3.5. Right: shows excitation at 310 nm where **TbTb·1** is set to pH = 8.

Energy Transfer investigations

The energy transfer rate from Tb(III) to Eu(III) can be estimated by comparing the photophysics of Tb(III) as observed in **TbTb·1** and **TbEu·1**. Figure S29 shows the absorbance spectra at pH 8 and 3.5, demonstrating that the pH-speciation is identical for both compounds.

Figure S30 and S31 show the excitation and emission spectra of **TbEu·1** demonstrating that the Tb-centered emission is nearly identical to the **TbTb·1** compound, as shown in the main manuscript. However, when excited at 350 nm, a very weak Eu(III) emission signal is observed. Multiplying this signal by 50 times provides comparable intensity to the Tb(III) emission when excited at 310 nm. Although the 350 nm excitation wavelength provides the brightest Eu(III) emission, it is found that the Tb(III) emission still dominates the emission spectra.

Figure S32 shows the normalized excitation spectra of **TbTb·1** (probed for Tb(III) emission), **TbEu·1** (probed for Eu(III) emission), and **EuEu·1** (probed for Eu(III) emission). The shapes of the excitation spectra for Tb(III)-based emission and Eu(III)-based emission are different. Figure S33 compares the spectra for the **EuEu·1** compound with the excitation and emission spectra of the carboxylic acid groups, as probed in the **GdGd·1** compound. The shape and positions of the bands indicate that Eu(III) based emission is the result of the carboxylic acids acting as antennas and not the aminophenol which acts as an antenna for Tb(III) emission.

Figure S33 shows the decay curves for Tb(III) luminescence in **TbTb·1**, **TbGd·1**, and **TbEu·1**. Following excitation both through the aminophenol and directly into Tb(III). The Eu(III) decay lifetime after excitation into 350 nm is also shown for the **EuEu·1** compound. The rate of terbium($^5D_4$)-to-europium energy transfer can be estimated based on the lifetimes fitted for these decay curves.

The lifetime of Tb(III) in **TbTb·1** ($\tau(\mathrm{Tb})_{\mathrm{TbTb}\cdot 1}$) and in **TbEu·1** ($\tau(\mathrm{Tb})_{\mathrm{TbEu}\cdot 1}$) are defined by Eq. S3 and S4 respectively.

$$\tau(\mathrm{Tb})_{\mathbf{TbTb\cdot 1}} = \frac{1}{k_{\mathbf{Lum}}+k_{\mathbf{Q}}} \qquad \text{Eq. S3}$$

$$\tau(\mathrm{Tb})_{\mathbf{TbEu\cdot 1}} = \frac{1}{k_{\mathbf{Lum}}+k_{\mathbf{EuTransfer}}+k_{\mathbf{Q}}} \qquad \text{Eq. S4}$$

Where k_{Lum} is the rate of luminescence, $k_{EuTransfer}$ is the rate of terbium($^5D_4$)-to-europium energy transfer, and k_Q is the rate of all other quenching. Assuming k_Q and k_{Lum} are the same in both compounds the $k_{EuTransfer}$ can be calculated by Eq. S5.

$$k_{\mathbf{EuTransfer}} = \frac{1}{\tau(\mathrm{Tb})_{\mathbf{TbEu\cdot 1}}} - \frac{1}{\tau(\mathrm{Tb})_{\mathbf{TbTb\cdot 1}}} \qquad \text{Eq. S5}$$

For direct excitation $\tau(\mathrm{Tb})_{\mathbf{TbTb\cdot 1}}$ and $\tau(\mathrm{Tb})_{\mathbf{TbEu\cdot 1}}$ gives 2.54 ms and 1.66 ms respectively and for antenna based excitation they give 2.53 ms and 1.73 ms. The rate of terbium($^5D_4$)-to-europium energy transfer is thus calculated to be 178 s^{-1} or 209 s^{-1} depending on the method. We provide 200 s^{-1} in the main manuscript.

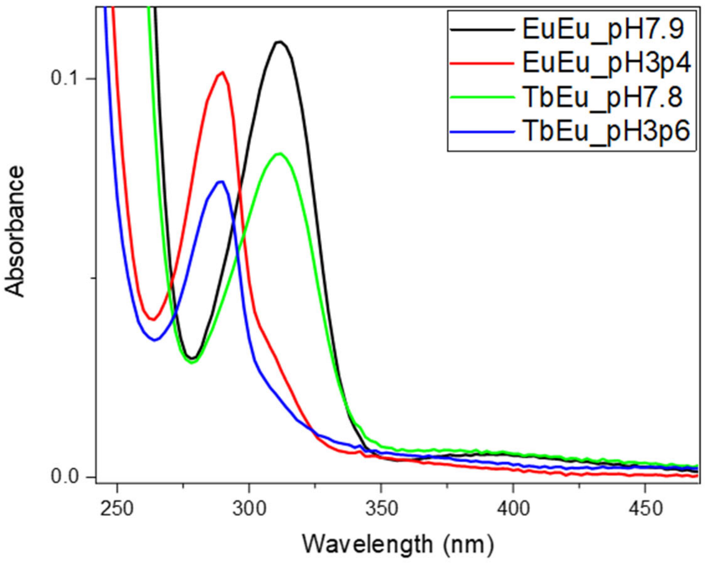

Figure S29: Absorbance spectra of **EuEu·1** and **TbEu·1** at pH 7.9 and 3.5.

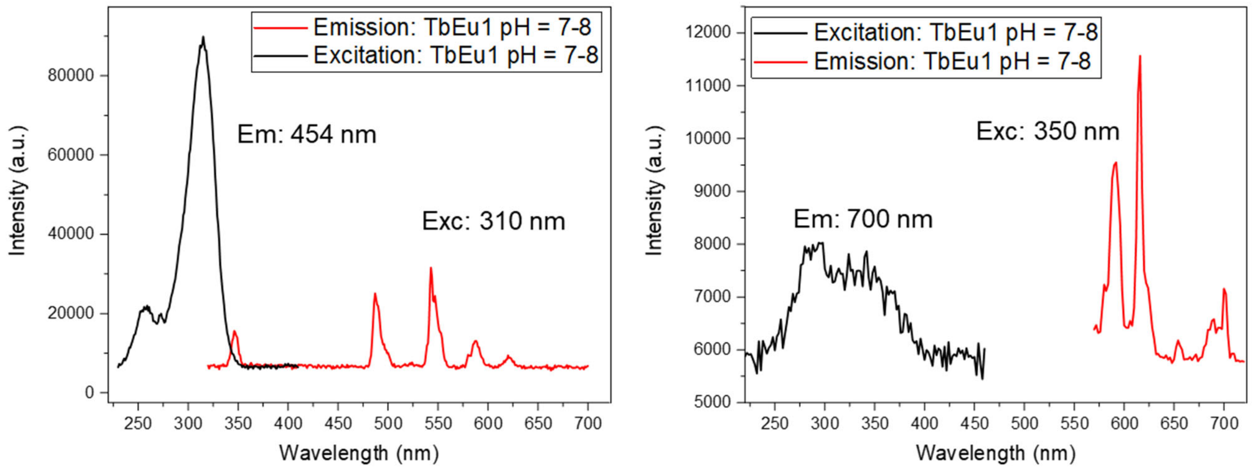

Figure S30: Excitation and emission spectra following Eu(III) (right) and Tb(III) (left) emission in **TbEu·1**

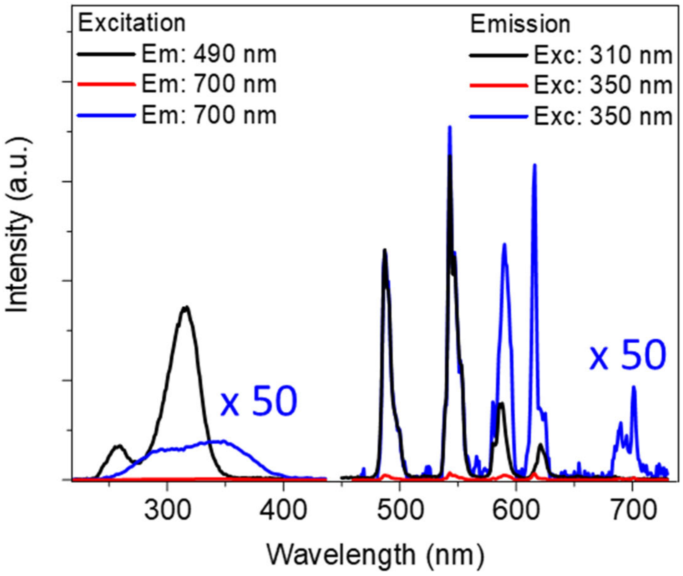

Figure S31: Excitation and emission spectra of **TbEu·1** at pH = 7.9. Excitation is collected for terbium centred luminescence at 490 nm and europium centred luminescence at 700 nm. Terbium emits most efficiently after excitation at 310 nm and europium emits most efficiently after excitation at 350 nm. The europium emission is comparatively diminishing with identical instrumental settings and the europium-based spectra have been multiplilied by 50 to highlight the differences.

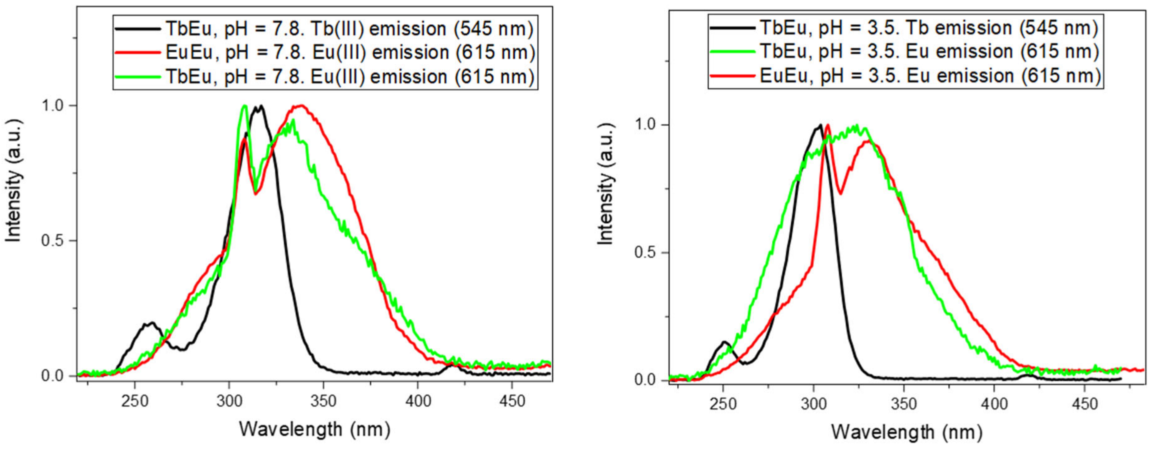

Figure S32: Normalized excitation spectra **EuEu·1** and **TbEu·1** at pH 7.8 (left) and 3.5 (right). **TbEu·1** is recorded from emission from Tb(III) and Eu(III) at 545 and 615 nm respectively and **EuEu·1** is only recorded from Eu(III) emission at 615 nm.

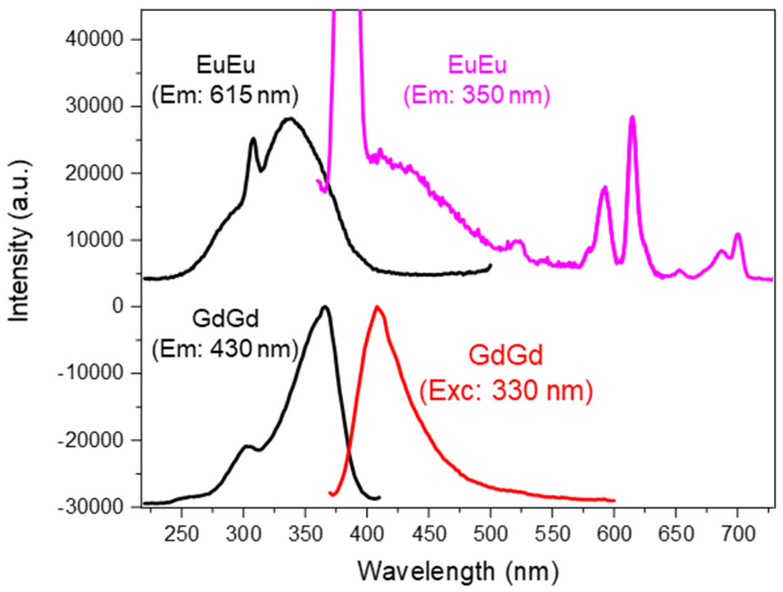

Figure S33: Excitation and emission of **EuEu·1** (top) measured at 615 and 350 nm respectively focusing on the transitions involved Eu(III). Excitation and emission of **GdGd·1** (top) measured at 430 and 330 nm respectively focusing on the transitions involved with the phosphorescence of the carboxylic acids.

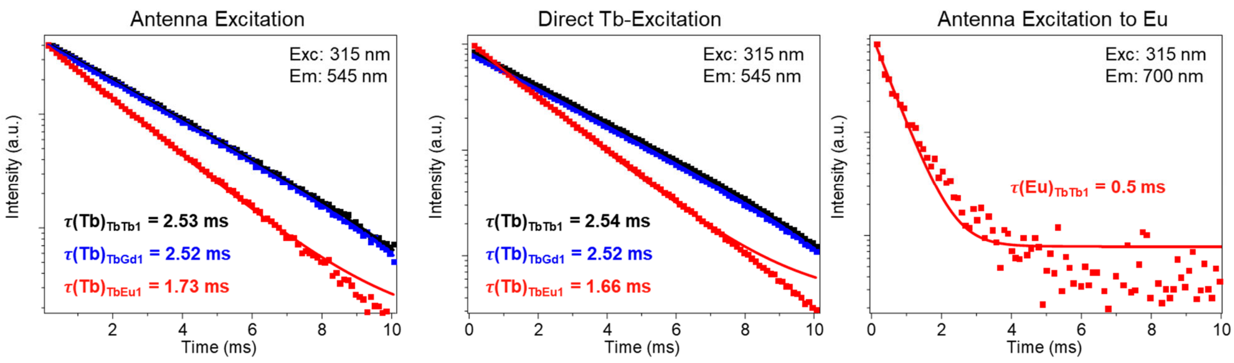

Figure S34: Time-resolved emission profiles and mon-exponential fits of **TbTb·1**, **TbGd·1**, and **TbEu·1** following excitation in the antenna and the Tb(III) excited state manifold monitored with Tb(III) centred emission, and following excitation in the antenna monitored with Eu(III) centred emission. The Eu(III) centred emission is not observable following excitation in the Tb(III) and Eu(III) excited stated manifold.

References

[1] A. Krezel, W. Bal, *J Inorg Biochem* **2004**, *98*, 161-166.

[2] a. C. H. Simms, D. Kovacs, L. Hacker, E. T. Sarson, D. Sokolova, K. E. Christensen, A. Khrapichev, L. A. W. Martin, K. Vincent, S. J. Conway, E. M. Hammond, M. J. Langton, S. Faulkner, *Chemistry – A European Journal*, *n/a*, e202404748; b. M. P. Placidi, L. S. Natrajan, D. Sykes, A. M. Kenwright, S. Faulkner, *Helvetica Chimica Acta* **2009**, *92*, 2427-2438.

[3] a. W. D. Horrocks, Jr., D. R. Sudnick, *Accounts of Chemical Research* **1981**, *14*, 384-392; b. W. D. Horrocks, Jr., D. R. Sudnick, *Journal of the American Chemical Society* **1979**, *101*, 334-340.

[4] M. P. Tosi, F. G. Fumi, *Journal of Physics and Chemistry of Solids* **1964**, *25*, 45-52.
